# Maternal Plasma Metabolic Profile Demarcates a Role for Neuroinflammation in Non-Typical Development of Children

**DOI:** 10.3390/metabo11080545

**Published:** 2021-08-18

**Authors:** Rebecca J. Schmidt, Donghai Liang, Stefanie A. Busgang, Paul Curtin, Cecilia Giulivi

**Affiliations:** 1 Department of Public Health Sciences, School of Medicine, University of California Davis, Davis, CA 95616, USA; rjschmidt@ucdavis.edu; 2 Medical Investigation of Neurodevelopmental Disorders (MIND) Institute, School of Medicine, University of California Davis, Sacramento, CA 95817, USA; 3 Gangarosa Department of Environmental Health, Rollins School of Public Health, Emory University, Atlanta, GA 30322, USA; donghai.liang@emory.edu; 4 Department of Environmental Medicine and Public Health, Icahn School of Medicine at Mount Sinai, New York, NY 10029, USA; stefanie.busgang@mssm.edu (S.A.B.); paul.curtin@mssm.edu (P.C.); 5 Department of Molecular Biosciences, School of Veterinary Medicine, University of California Davis, Davis, CA 95616, USA

**Keywords:** autism, non-typical development, metabolomics, prostaglandins, neuroinflammation, pregnancy, cord blood

## Abstract

Maternal and cord plasma metabolomics were used to elucidate biological pathways associated with increased diagnosis risk for autism spectrum disorders (ASD). Metabolome-wide associations were assessed in both maternal and umbilical cord plasma in relation to diagnoses of ASD and other non-typical development (Non-TD) compared to typical development (TD) in the Markers of Autism risk in Babies: Learning Early Signs (MARBLES) cohort study of children born to mothers who already have at least one child with ASD. Analyses were stratified by sample matrix type, machine mode, and annotation confidence level. Dimensionality reduction techniques were used [i.e, principal component analysis (PCA) and random subset weighted quantile sum regression (WQS_RS_)] to minimize the high multiple comparison burden. With WQS_RS_, a metabolite mixture obtained from the negative mode of maternal plasma decreased the odds of Non-TD compared to TD. These metabolites, all related to the prostaglandin pathway, underscored the relevance of neuroinflammation status. No other significant findings were observed. Dimensionality reduction strategies provided confirming evidence that a set of maternal plasma metabolites are important in distinguishing Non-TD compared to TD diagnosis. A lower risk for Non-TD was linked to anti-inflammatory elements, thereby linking neuroinflammation to detrimental brain function consistent with studies ranging from neurodevelopment to neurodegeneration.

## 1. Introduction

Currently, approximately 1 in 54 children has been identified with Autism Spectrum Disorders (ASD) in the United States according to estimates from the CDC’s Autism and Developmental Monitoring (ADDM) Network [1]. Prevalence of ASD has been increasing in recent decades and estimates worldwide are highly variable [2]. ASD occurrence is higher in males [1], and in younger siblings of individuals diagnosed with ASD, at nearly 1 in 5 [3]. Often clinical diagnosis is not made in children before significant neurological symptoms are evident, thus there is an immediate need for early detection of processes that are altered and could be used as markers for risk and to potentially inform effective interventions to allow them to develop life to their fullest.

To fulfill this immediate need, metabolic profiling performed on readily-accessible body fluids, such as serum, plasma, blood, urine or saliva, is one of the most important techniques that provides a complete view of the metabolic status and uncovering metabolic perturbations in pathways [4,5,6] for diagnosis of many diseases [7], including metabolic disorders [8], motor neuron disease [9], Parkinson’s disease [10] and Alzheimer’s disease [11], as well as chemical toxicity and aging [12,13,14,15]. There are few studies to date that have examined pre- and peri-natal metabolic pathways in relation to later development of ASD [16,17,18], with limited insights isnto the link between key metabolites, their role in intermediary metabolism and recurrent risk in families affected by autism. To this end, in this study, we assessed the metabolome from 3rd-trimester maternal plasma and from umbilical cord plasma samples in association with clinical classification of ASD or non-typical development (Non-TD) compared to typical development (TD) in high-familial-risk younger siblings of children with ASD. We used a metabolome-wide association approach to assess single metabolites as well as dimensionality reduction techniques including principal component analysis (PCA) and the random subset implementation of weight quantile sum regression (WQS_RS;_ [19,20]) to account for the large number of predictors in relation to the number of subjects. The aim was to provide a complete view of the status of intermediary metabolism with the potential of uncovering perturbations in metabolic pathways [5,6] associated with the diagnosis of ASD or classification of Non-TD.

## 2. Results

### 2.1. Participant Characteristics and Covariate Selection

Two-hundred and sixteen mother–child pairs provided a sample for at least one of the following timepoints: 1st trimester, 2nd trimester, 3rd trimester, and/or delivery. The current analysis included only those participants who contributed with either a 3rd-trimester plasma sample and/or a cord plasma sample at delivery (*n* = 209). A covariate-only model was used to identify covariates that differed across clinical classification (all 3 groups) based on a *p*-value less than 0.20. The covariates identified for inclusion in final models were child’s sex, gestational age at delivery (in weeks), and maternal body mass index (BMI) before pregnancy. Child’s race was also included in the final models. Participants missing any of these covariates or a final outcome (*n* = 10) were removed from further analyses. Table 1 shows the demographic characteristics of the final cohort with no missing covariates, and stratified by outcome group (*n* = 199). The final cohort included 115 children clinically classified as TD, 27 children classified as Non-TD, and 57 diagnosed as having ASD. Children with ASD were more likely to be male and had longer mean gestational age at delivery compared with children classified as TD. Mothers of children with Non-TD and ASD had higher mean pre-pregnancy BMI compared to mothers of TD children. Of the 199 participants, 184 contributed a 3rd-trimester plasma sample and 142 contributed a cord blood plasma sample.

### 2.2. Metabolomic Analyses

#### 2.2.1. Full Feature Metabolome Set

After removing metabolites with less than 80% detection, the full feature set included annotated and unannotated metabolites, which ranged from 4553 to 6278 metabolites depending on the sample matrix and mode of the LC/HRMS (Supplementary Materials Figures and Tables summarize the outcomes and statistical analysis derived from each matrix and mode).

In initial analyses, we constructed discrete logistic models following a metabolome-wide association study (MWAS)-type analysis to evaluate associations between individual metabolites and diagnostic status, while controlling for covariates described previously. For each mode (positive and negative), measured from both maternal and cord plasma samples, there were many unannotated features identified in single-metabolite models that either significantly increase or decrease the association with risk of ASD and Non-TD compared to TD while controlling for child’s sex and race, gestational age in weeks, and mother’s pre-pregnancy BMI (Figures S1–S4). For all associations, none remained significant after correcting for multiple comparisons.

Next, we used PCA to identify PCs that explain the variance within the metabolite data. Using metabolites identified from the positive mode of maternal plasma, the mean PC13 score was significantly lower in ASD children compared to TD while controlling for covariates (Figure S5a). The top ten contributing components are displayed in Figure S5b; however, as not one of them is annotated, their present biological significance remains unknown. From PCA of metabolites from the negative mode of maternal plasma, we found that PC1, PC6, and PC12 were significantly different between ASD and TD and/or Non-TD and TD (Figure S6a) with the top contributing components displayed in Figure S6b. Two of the top 10 components from PC1 were also identified as significantly related to a decrease in odds of Non-TD in single-metabolite analyses (palmitic acid and linoleic acid). Glutamine was the only annotated metabolite that was a top component of PC12. All other top 10 components for PC1, PC6 and PC12 were unannotated and thus their biological significance is unknown. No PCs were identified as being significantly different using the PCA of metabolites identified from positive mode cord plasma (results not shown). Lastly, from the PCA of metabolites identified from negative mode cord plasma, we identified PC12 as having a mean score significantly higher in children with ASD compared to TD (Figure S7a) with the top contributing components displayed in Figure S7b. Similar to the other significant PCs indicated above, none of the top 10 components were annotated.

As an alternative dimensionality reduction strategy to the PCA, we employed a supervised technique to identify the effect of a single index composed of all metabolites on diagnosis while controlling for covariates. By using this index, we can identify which metabolites most heavily weigh the effect if the index is significantly related to the outcome. For the metabolites identified in the positive mode of maternal plasma, there were no associations between the index and either outcome (*p*-values range between 0.10 and 0.37). For metabolites identified in the negative mode of maternal plasma, the mixture was significantly reduced in Non-TD compared to TD [Odds Ratio (OR) = 0.31, 95% Confidence Interval (CI) = 0.10–0.93] such that for every one unit increase in the mixture index, the odds of being Non-TD were 0.31 times the odds of being TD. The top 10 weight components of this mixture effect are displayed in Table S2. Other negative mode plasma indices were not significantly related to diagnosis (*p*-values range between 0.19 and 0.87; Table S3). The indices of metabolites identified from both the positive and negative modes of cord plasma were not significantly associated with either outcome (*p*-values range between 0.30 and 0.98; Table S3).

#### 2.2.2. Annotated Metabolome Set

The confidence level 1 annotated subset included between 37 and 43 metabolites with at least 80% detection, reducing the number of comparisons compared to the full feature set (Table S1 summarizes the measures derived from each matrix and mode).

Like the full feature set, discrete logistic single-metabolite models, controlling for child’s sex and race, gestational age in weeks, and mother’s pre-pregnancy BMI, were analyzed for the annotated-only subsets. Four annotated metabolites detected in the positive mode of maternal plasma increased risk of ASD (*O*-phosphoethanolamine, indole, betaine, and serine) and four metabolites (*N*-acetylglutamic acid, citrulline, acetylcarnitine, and cystathionine) decreased the risk of Non-TD while controlling for covariates; however, none of these associations remained significant after adjusting for multiple comparisons (Table S4; Figure S8). In single-metabolite models from the negative mode of maternal plasma, two metabolites, norvaline and mevalonic acid, significantly increased the risk of ASD compared to TD (OR [95% CI]: 1.53 [1.07–2.26] and 1.68 [1.09–2.76], respectively). Eleven metabolites significantly decreased the risk of being diagnosed as Non-TD compared to TD (ORs ranging from 0.28 to 0.64), and one metabolite, gulonolactone, increased the risk of Non-TD (OR [95% CI]: 2.70 [1.18–7.57]). Eight of these metabolites continued to be significantly negatively associated with Non-TD after FDR correction (Table 2; Figure S9).

One annotated metabolite in the positive mode of cord plasma significantly decreased the risk of ASD, two metabolites significantly decreased the risk of Non-TD, and one metabolite significantly increased the risk of ASD and Non-TD each compared to TD (Table S5; Figure S10). One metabolite identified in the negative mode of cord plasma significantly decreased the risk of Non-TD compared to TD. However, none of these associations from cord plasma, regardless of mode, remained significant after FDR correction (Table S6; Figure S11).

PCA was run on the annotated-only set and mean PC scores were compared between diagnostic groups while controlling for covariates. Mean scores of PC2, PC8, and PC13, identified from the PCA of metabolites in the positive mode of plasma were considered significantly different for ASD, Non-TD or both compared to TD (Figure S12a). Specifically, PC2 and PC13 were significantly associated with Non-TD (NT) compared to TD (β = 0.88, *p* = 0.017 and β = 0.55, *p* = 0.019, respectively), while PC8 was significantly associated with ASD compared to TD (β = 0.41, *p* = 0.052). Components with high contributions tended to overlap with metabolites identified in single-metabolite models (Figure S12b; see Table S3 for comparison). The mean score of PC1 and PC8, identified from the PCA of metabolites in the negative mode of plasma, were significantly different between Non-TD and TD (β = −2.16, *p* = <0.001 and β = 0.60, *p* = 0.017, respectively; Figure S13a). Top contributing components are displayed for each PC (Figure S13b). The mean scores of PC4, PC5, and PC11, identified from the PCA of metabolites in the positive mode of cord plasma, were significantly different between Non-TD and TD (β = −0.98, *p* = 0.010; β = 0.76, *p* = 0.046; and β = −0.61, *p* = 0.050, respectively; Figure S14a). Top contributing components are displayed for each PC (Figure S14b). Lastly, the mean score of PC6, derived from PCA of metabolites in the negative mode of cord plasma, was significantly reduced in Non-TD compared to TD (β = −1.16, *p* = 0.001; Figure S15a) with the top contributing components of PC6 being displayed in Figure S15b.

WQS_RS_ was used to estimate weights with only the subset of annotated features and the association between the weighted index and each diagnostic group, controlling for covariates, was tested. The WQS_RS_ indices, using metabolites from the positive mode of plasma, were not significantly associated with either diagnostic group (*p*-values range from 0.28–0.94; Table S7). Although no WQS_RS_ indices of metabolites from the negative mode of maternal plasma were significantly related to either outcome, there was a negative trend between the mixture and Non-TD (OR = 0.56, 95% CI = 0.30–1.04) and the top ten weight components are displayed in Table S6. For other indices, *p*-values ranged from 0.62–0.92. Lastly, no WQS_RS_ indices of metabolites from cord plasma, regardless of mode, were significantly associated with either diagnostic group (*p*-values range from 0.26–0.78; Table S7). Models assessing metabolites from positive cord plasma on risk of NT did not converge (not shown).

### 2.3. Fatty Acids in Prostaglandin Pathways

**Figure 1:**
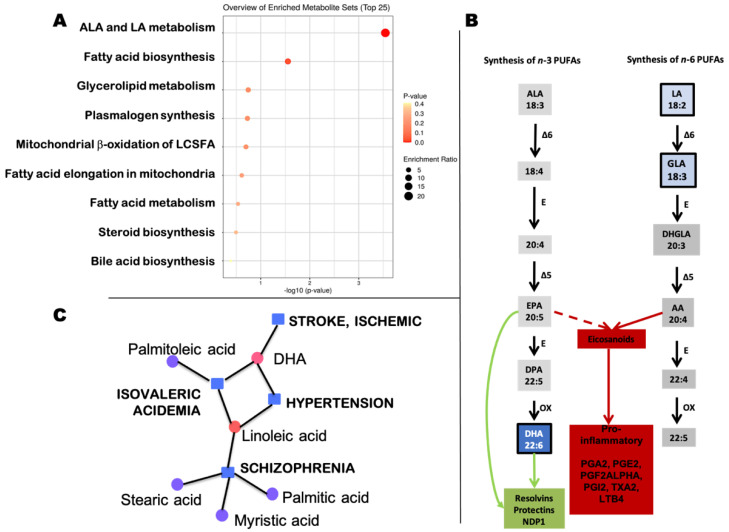
Overview of metabolites identified as relevant to decrease the Non-TD risk in the context of the biosynthesis of fatty acids and eicosanoids. (**A**). Pathway enrichment analysis performed with significant metabolites from Table 2. Analysis was performed by utilizing the KEGG database. (**B**). Metabolites in grey were not identified in this study; those in blue boxes were identified and the blue shade represents the value of the OR from Table 2. Abbreviations: ALA, alpha-linolenic acid; LA, linoleic acid; EPA, eicosapentaenoic acid; DPA, docosapentaenoic acid; DHA, docosahexaenoic acid; AA, arachidonic acid; DHGLA, dihomo-gamma-linolenic acid; MUFAs, monounsaturated fatty acids; PUFAS, polyunsaturated fatty acids; SFAs, saturated fatty acids; E, elongase; desaturases are indicated as delta followed by the bond affected; OX, peroxisomal beta-oxidation. Eicosanoids with anti-inflammatory properties (resolvins, protectins) are indicated with thin green arrows, whereas pro-inflammatory (prostanglandins of the series 2 and LTB4) are indicated with thick red arrows. (**C**). Metabolite–disease analysis was performed in MetaboAnalyst.

Seven metabolites identified in the negative mode of maternal plasma—after adjusting for baby’s sex, maternal pre-pregnancy BMI, gestational age and race (Table 2)—significantly decreased the risk of Non-TD compared to TD. Five of these metabolites are part of the biosynthesis of unsaturated fatty acids, and more specifically the pathway that includes ALA and linoleic acids (Figure 1A; FDR = 0.0281). While both pathways, the one that starts with ALA (prostaglandins series-3) and the one that starts with linoleic acid (prostaglandins series-2), may generate eicosanoids leading to proinflammatory prostaglandins, the former also gives rise to less proinflammatory mediators such as resolvins, protectins and NDP1 (i.e., oxylipins [21]; Figure 1B). It has been demonstrated that an imbalance in the ratio of *n*-6/*n*-3 PUFAs during early developmental stages may result in metabolic reprogramming, thereby influencing the susceptibility of adults to diverse diseases [22]. To this end and to visualize the impact of the differential metabolites in these two pathways, we plotted their predictive value on their activation (Figure 1B). The comparison of the average OR of the *n*-3 series (LA and GLA) vs. that of the *n*-6 series (DHA) showed a more important role in the Non-TD risk for DHA than that of those in the *n*-3 series, suggesting that a balance between pro- and less inflammatory compounds are relevant for the risk of Non-TD. Moreover, when a metabolite–disease network analysis was performed with the 7 metabolites from Table 2, two conditions related to developmental delay, speech delay, and disruptive behavior showed significance, i.e., schizophrenia (degree of 4 and betweenness of 21) and isovaleric acidemia (degree of 3 and betweenness of 13; Figure 1C).

## 3. Discussion

We face many challenges if we are to more fully understand the biological factors that affect ASD and/or Non-TD risk within natural populations. First, despite major advances in our understanding of the molecular basis of ASD, we are still far from a comprehensive understanding of the causal mechanisms, both molecular and environmental, which influence ASD risk and morbidity. ASD, as a spectrum disorder, may present as an isolated set of symptoms or with multiple comorbidities (e.g., intellectual disability, developmental delay, epilepsy, gastrointestinal complications, cardiac problems, and immune disorders [23]) with a broad heterogeneity that is still apparent even in the settings of identical genetic backgrounds (e.g., monozygotic twins discordant for co-morbidities), underscoring the complexity of understanding ASD on the molecular level. Despite this, it is clear that ASD has heritable component intertwined with non-heritable ones (shared environment) with siblings of ASD subjects usually having a ten-fold higher than population average risk of developing ASD themselves [3]. Further, concordance between monozygotic twins ranges from 30% to 99%, and the overall heritability is estimated between 0.5 and 0.8 [24,25,26,27,28]. The overarching challenge has been that despite measures of heritability pointing to a strong genetic component for ASD as it is for most complex traits in human populations, the failure to find causative alleles for traits that often have a high heritability is part of the larger ‘missing heritability’ problem in humans, where the sum of all the genetic effects associated with highly heritable traits is typically far less than the heritability measured (reviewed in [29]). This is further illustrated by GWAS which usually can explain only a small fraction of that heritability [30,31], leading to the aforementioned ‘missing heritability’ problem. One common hypothesis is that this shortfall is due to traits being shaped by many common alleles with effect size too small to detect and by large-effect alleles that are too rare to detect [32]. Second, and connected with the previous point, the environment can have a significant effect on ASD risk, but determining the specific impact of slight changes in the environment, as well as gene-by-environment interactions, on ASD risk has proven difficult [33,34,35]. This has shifted the focus to find significant ASD-associated transcriptome differences across genes in tissues not necessarily related to CNS that may capture some of the variance of the ASD phenotype [36,37,38,39,40,41,42,43,44,45]. However, growing evidence suggests mRNA levels do not capture all variance in phenotypes because they do not necessarily correlate with protein expression levels [46,47], reinforcing the need to highlight that proteins make up the enzymes that catalyze different biological reactions, and metabolites that are the building blocks of structural elements and biochemical pathways and act as modulators of metabolic fluxes at key rate-limiting steps. These challenges suggest that examining genes and genetic variation alone may not provide a complete picture of the molecular mechanisms that influence ASD risk. Genes shape phenotypes by working through a complex network of various molecular (‘omic’) domains that exist between genotype and phenotype. Then, investigating these functional molecular domains will help elucidate the causal mechanisms of ASD as well as the endophenotypes because (specifically metabolites) capture variation seen not only in genes but also in the environment, thereby allowing us to understand the biological impact of the environment on ASD. In addition, the interaction between metabolites per se is critical for regulating the functions that maintain cellular homeostasis. By looking not only at changes in mean levels of metabolites but also in the way that abundances of these molecules correlate with one another in larger networks, we can generate new and potentially powerful hypotheses about ASD risk. Networks—such as the one shown in Figure 1C—consist of a set of ‘nodes’ (here, metabolites) connected to one another through “edges”. The edges in biological networks can be defined either by directional biochemical interactions (precursor-product link), nondirectional biochemical interactions (protein-protein interaction) or more commonly, by expression correlations (e.g., correlation of concentrations of two or more metabolites). From a network perspective, the critical changes that occur with ASD risk may not be changes in concentrations per se (i.e., removing or adding a node in the network), but rather changes in edges connecting different nodes. For example, one could imagine two metabolites that have the same concentrations in TD and ASD, but whose correlation changes with age or other conditions (e.g, diet; example [48]). Much of the literature on biomarkers of ASD and/or IDD has focused on the search for individual molecules, or statistical combinations of molecules, which are predictive of ASD-related morbidity or diagnosis [16,49,50,51,52,53,54,55,56,57,58,59,60,61,62,63,64,65,66,67,68,69,70,71,72,73,74,75,76,77,78,79,80,81,82,83,84,85,86,87]. However, few metabolomic studies have moved beyond individual, isolated biomarkers, focusing instead on metabolic pathway analysis, in which whole metabolic pathways are analyzed for significant changes. To cite a few examples, at the prenatal level, Ritz et al. [16] showed differences in several metabolic pathways, including glycosphingolipid biosynthesis and metabolism, *N*-glycan and pyrimidine metabolism, bile acid pathways and, importantly, C21-steroid hormone biosynthesis and metabolism in maternal mid-pregnancy serum samples from women residing in California and whose children later developed ASD. Nolin et al. [88] reported deficits in the serine biosynthetic pathway as a main source of anaplerosis for the Krebs’ cycle in amniotic fluids from women carrying a fetus with a *FMR1* premutation vs. noncarriers. In ASD children, Xu et al. [50] reported altered taurine and hypotaurine, Phe, Arg, and Pro metabolism in both plasma and urine samples. Liang et al. [63] reported deficits in urinary amino acid or lipid metabolism as well as the Trp-kynurenine pathway in children with ASD. Metabolomic profiles of 30 healthy children, 15 with and without regression each, showed differences between typically developing and children with ASD involving mainly amino acid, lipid and nicotinamide metabolism with subtle differences between the two regression groups (Arg and Glu pathways) [70]. Through a targeted urine metabolomics of Lebanese children affected by autistic disorder, changes in Tyr, 2-hydroxybutyrate, creatine and Glu pathway were reported with implications for amino acids, carbohydrates and oxidative stress pathways [77]. Italian children with ASD showed urinary metabolites belonging to the Trp and purine metabolic pathways along with vitamin B6, riboflavin, aromatic amino acid biosyntheses, pantothenate and CoA, and pyrimidine metabolism [80]. Previous metabolomics analysis of plasma in children with ASD, Down syndrome, and idiopathic developmental delays compared to typically developing children found perturbation in one-carbon metabolism pathways for children with ASD and Down syndrome [72].

Deciphering entire pathways that are differentially regulated in ASD leads to new hypotheses about the underlying biological processes that may be influencing its development. In this light, our study focused on the evaluation of the biological products of proteins (metabolites) via global metabolomic profiling and connect them through a thorough networked biological pathway analysis. We suggest here that this approach can provide a more robust method to discover causal mechanisms underlying ASD risk. Here, using a non-targeted maternal and cord plasma metabolomics screening approach in a clinically well-characterized cohort of subjects, provided results indicating important insights. For the full feature set, dimensionality reduction strategies were useful in identifying metabolites related to diagnostic group compared to single-metabolite analyses which suffer from multiple comparisons. Single-metabolite analyses of annotated features subsets also suffered from the burden of multiple testing, although some metabolites remained significantly associated with diagnosis after applying the FDR correction. Although none of the annotated metabolites were significantly associated with ASD after FDR corrections, the four maternal metabolites most associated with increased ASD (*O*-phosphoethanolamine [89], indole [80,90], betaine [79,91], and serine [53,72,87,88,92,93,94]) were in pathways previously implicated in ASD etiology. Further, acetylcarnitine, one of the most associated maternal single metabolites for Non-TD (positive node), has also been previously identified as a metabolic marker of ASD [95].

Between the full feature and annotated-only sets, the findings consistently indicate that metabolites identified in the negative mode of maternal plasma were more often associated with a decreased risk of Non-TD compared to TD. Several metabolites were identified across all analysis strategies, providing further evidence of their importance. For example, the unsaturated fatty acids DHA, LA, GLA and palmitoleic and the saturated myristic, palmitic and stearic acids appear to be important for distinguishing diagnosis of Non-TD from TD in all analyses of metabolites identified in the negative mode from 3rd-trimester maternal plasma.

When these metabolites in maternal plasma were analyzed in terms of their biological relevance, several pathways were identified. Although it could be argued that plasma levels of metabolites do not represent those in brain, it is important to highlight that unesterified fatty acids readily cross the blood–brain barrier into the brain [96], representing the major peripheral form that mirrors PUFA metabolism in the brain. Additionally, several examples from the literature link changes in these fatty acids with diseases. It has been reported that decreases in red blood cells’ membrane PUFAs from subjects with schizophrenia correlates with the degree of demyelination in brain white matter [97]. Lower levels of free fatty acids were reported in post-mortem brain samples from subjects affected with unipolar and bipolar depression [98]; lower levels of oleic and arachidonic acids were observed in frontal cortex from subjects with Alzheimer’s disease [99] and with Parkinson’s disease [100], respectively.

Notably, the OR for Non-TD risk is lower in the *n*-3 series (DHA) vs. that of the *n*-6 series (LA, GLA). This finding is consistent with those reported in another study in which children with ADHD and ASD presented lower levels of EPA, DHA and arachidonic acid and high ratio of *n*-6/*n*-3 PUFAs and both of these ratios correlated significantly with behavioral scores (ATBRS, TOVA, CARS; [101]). This is highly relevant as *n*-3 PUFAs foster neuronal activity [102] counteracting memory deficits in aged mice [103], enhance mitochondrial function [104], increase the expression of glucose transporters GluT1/2 influencing neurotransmissions [105], ameliorate spatial memory in rats by increasing the expression of subtypes of endocannabinoid receptors [106], increase the expression of transcription factors involved in learning and memory [107], improves childhood neurodevelopment [108], and increase brain function and decreased tau phosphorylation in a mouse model of Alzheimer’s disease with enhanced endogenous production of *n*-3 PUFA [109].

Neuroinflammation is recognized as the inflammatory reaction occurring in the central and peripheral nervous systems, primarily caused by environmental pollutants and toxicants, trauma, and autoimmune responses, among others [110] and the occurrence of various neurological diseases is closely related to neuroinflammation [111]. Consistent with this view, an increased pro-inflammatory status seemed to increase the Non-TD risk as more pro-inflammatory prostaglandins are generated by the n-6 series than those from the n-3 series [112] (Figure 1B). In this regard, PGE2 in neural injury in Alzheimer’s disease is well documented, and includes modulation of protein-lipid interactions, trans-membrane and trans-synaptic signaling [113]. Moreover, PGE2 levels in CSF have been identified as one of the key pathways linked to Alzheimer’s disease severity [114]: PGE2 is higher in patients with mild memory impairment, but lower in those with more advanced Alzheimer’s disease [115]. Two recent reviews [116,117] highlight the role of inflammatory mediators (including prostaglandins), and as an extension that of neuroinflammation, on the development of an altered mother–child immune crosstalk during pregnancy which is critical for atypical neurodevelopment. Inflammatory mediators can alter not only critical functions of neurons and glia directly, but also affect the selective permeability of the blood–brain barrier providing a mechanistic link to altered brain development as well as the occurrence of epilepsy in ASD [118]. Therefore, appropriate interventions that control immune responses during pregnancy may have a positive impact on decreasing autism diagnosis risk.

The significantly lower Non-TD risk with myristic, palmitic, stearic and palmitoleic acids deserves further discussion considering the role of these factors in axonogenesis, neuron differentiation, and carbohydrate utilization. Axonogenesis requires the de novo synthesis of monounsaturated fatty acids (such as palmitoleic acid) based on the evidence that brain-derived neurotrophic factor (BDNF) promotes both axonogenesis during brain development [119] while selectively increasing intracellular levels of palmitoleic acid and SCD1 [120], and that SCD-1 is highly expressed in axotomized neurons of the regenerating facial and hypoglossal nucleus [121]. More recently, a role for palmitoleic acid as an insulin-sensitizing lipokine has been proposed (although still controversial), suggesting that lower levels of this fatty acid may limit carbohydrate utilization. Although blood metabolite levels can change in response to diurnal/circadian hormonal levels, diet, stress, underlying (not diagnosed) conditions, among others [122,123,124,125,126,127], none of those identified as having a significant time-of-day variation were among those found to increase the OR of a Non-TD diagnosis (acylcarnitines, lysophospholipids, bilirubin, corticosteroids, and amino acids [127]. Further, in analysis for a subset of 175 with maternal samples, time since last meal or snack at the time of the blood draw, illnesses in the previous 48 h, and storage time did not differ across child outcomes, making these unlikely confounders. Future analyses should collect and control for these variables to rule out potential for residual confounding.

Metabolomics analysis may expedite additional diagnosis while allowing for the possibility to identify sources of phenotypic heterogeneity within the broad umbrella of Non-TD diagnosis. A strength of this study is in utilizing metabolomics to detect changes in a broad variety of metabolites that reflect the complexity of metabolic networks altered in Non-TD vs. TD. Future studies will need to assess the progression of changes in the identified pathways to shed light on the mechanisms contributing to the Non-TD morbidity. Future research should investigate maternal and neonatal metabolic changes in relation to upstream prenatal exposures, with a focus on those that are modifiable.

## 4. Materials and Methods

### 4.1. Study Design and Population

Mothers who have previously given birth to a child with autism spectrum disorder (ASD) were recruited into Markers of Autism risk in Babies: Learning Early Signs (MARBLES) [128] during pregnancy. Recruitment is primarily from lists of children receiving services for ASD through the California Department of Developmental Services in Northern California. The ASD of the older sibling is clinically confirmed. The younger-born siblings of children with ASD are at high familial risk for ASD and other Non-TD [3]. These analyses include additional participants at high familial risk due to the mother having multiple siblings diagnosed with ASD, but without an older ASD-diagnosed sibling. Blood samples were collected by certified phlebotomists during the 3rd trimester of pregnancy, and umbilical cord blood was collected at delivery. Samples were put on ice immediately, processed as soon as possible (within 4 h), and the plasma was stored at −80 °C until they were processed at Emory (only specimens without previous thaws were selected). The analytic sample for this study was derived from MARBLES-enrolled younger siblings who had a cord blood sample available and/or whose mothers had a 3rd-trimester EDTA plasma sample available, and who had received a final clinical assessment and outcome classification at 3 years old. The University of California, Davis Institutional Review Board and the California Committee for the Protection of Human Subjects approved this study and the MARBLES study protocols (IRB Protocol: 225645-70), which were conducted in accordance with the Helsinki’s guidelines. Neither data nor specimens were collected from children until written informed consent was obtained from their parents.

### 4.2. ASD and Non-TD Outcome Classification

Children were followed and assessed for ASD and other neurobehavioral and cognitive outcomes at 3 years of age at the MIND Institute. Development was assessed by trained, reliable examiners using the gold standard Autism Diagnostic Observation Schedule (ADOS) [129,130]. Cognitive function was measured with the Mullen Scales of Early Learning (MSEL) [131], which generates five subscale scores (gross motor, fine motor, expressive language, receptive language, and visual reception) and an overall early learning composite (ELC) score. Participants were classified into one of three outcome groups, ASD, typically developing (TD), and non-typically developing (Non-TD), based on a previously published algorithm that uses ADOS and MSEL scores [132,133] and that has shown good reliability, validity, and stability [134,135] for the ASD diagnosis at age 3 years in high-risk siblings of children with autism [133]. Children with ASD outcomes have scores over the ADOS cutoff and meet DSM-5 criteria for ASD. The Non-TD group was defined as children with low MSEL scores (i.e., two or more MSEL subscales that are more than 1.5 SD below average or at least one MSEL subscale that is more than 2 SD below average), elevated ADOS scores (i.e., within 3 points of the ASD cutoff), or both. Children classified as TD have all MSEL scores within 2.0 SD and no more than one MSEL subscale that is 1.5 SD below the normative mean and scores on the ADOS at least three or more points below the ASD cutoff [136]. Children classified as Non-TD or TD could develop other behavioral or neurodevelopmental abnormalities as they age [134].

### 4.3. Metabolomics Analysis

Metabolomic assays were run on plasma obtained from EDTA-supplemented blood samples from mothers during the 3rd trimester of pregnancy (maternal) and from cord blood (cord plasma) at delivery using liquid chromatography coupled with high-resolution mass spectrometry (LC/HRMS). Samples were analyzed in triplicate and metabolites were measured in hydrophobic interaction liquid chromatography (HILIC) Positive mode and C18 hydrophobic reverse-phase negative mode using established protocols [137,138]. For quality control purposes, at the beginning and end of each batch, we included two standards, NIST 19,501 and pooled human plasma (Equitech Bio). Raw data files were converted with ProteoWizard to mzML files using apLCMS and xMSanalyzer [139,140,141]. Metabolite annotations were scored according to criteria specified by the Metabolomics Standard Initiative (MSI) to denote the level of confidence in feature annotation [142]. Analyses were performed on the level 1 confidence subset in addition to the full feature set, stratified by HILIC positive and C18 reverse phase negative modes, herein referred to as positive and negative modes. Separate analyses were conducted for maternal and cord plasma samples. Metabolites with less than 80% detect were removed from further analyses. Missing values were replaced with the minimum of a specified metabolite divided by the square root of 2. To normalize across metabolites, values were log2 transformed and centered and scaled. Single-metabolite logistic regression models, with TD as the reference, were used to measure the effect of each metabolite on the diagnosis of ASD or Non-TD, respectively. Covariates were selected by testing a full covariate model with the multinomial outcome, keeping variables with a *p*-value less than 0.20. Final models were adjusted for child’s sex, child’s race (white or other), gestational age in weeks, and maternal body mass index (BMI) before pregnancy. To account for multiple comparisons, a false discovery rate (FDR) correction was applied to the raw *p*-values per stratum of sample matrix, mode, and feature set [143]. Metabolomic assays can result in hundreds to thousands of metabolites and as a result, adjusting for multiple comparisons in single-metabolite analyses can increase type I error. To reduce this burden, we employed unsupervised and supervised dimensionality reducing techniques. We employed an unsupervised approach, principal component analysis (PCA), to identify principal components (PCs) that explain the variability within the metabolite data. To evaluate the relevance of derived features to diagnostic status, PCs with an eigenvector >1 or >1% were isolated and examined in generalized linear models, controlling for the same covariates as previously described. For PCs with a mean score that significantly differed between ASD or Non-TD with respect to TD, the top contributing components are labeled either by their annotation; or, as their mass over charge ratio and retention time if the metabolite could not be annotated with confidence level 1. To test for the mixture effect of metabolites using a supervised dimensionality reduction technique, the random subset implementation of weighted quantile sum regression (WQS_RS_) was used [19]. This is a specialized application of WQS [20] which tests for a mixture effect among many components with complex correlations. Specifically, WQS_RS_ is useful when the number of predictors is greater than the number of subjects, which is the case with metabolomic data that can include hundreds to thousands of metabolites. Similar to the single-metabolite analyses, mixture effects were tested on separate binomial models for ASD and Non-TD relative to TD and were stratified by mode, sample matrix, and annotation level. Annotated-only models were run with 1000 subsets with 6 metabolites per subset and full feature models were run with 30,000 subsets with 10 metabolites per subset. Additionally, mixture models were run with both a positive and negative constraint to see if there were both harmful and protective mixtures related to the disease outcomes. A positive constraint more highly weighs components in the mixture that increase risk, whereas the negative constraint more highly weighs components that decrease risk.

### 4.4. Statistical Analyses

An alpha of 0.05 was the criterion for statistical significance. Statistical analyses were conducted with SAS 9.4 and R version 3.5.2 using the, *factoextra* package for PCA analyses. All analyses relevant to biological processes were performed with MetaboAnalyst by utilizing the KEGG database and those related to diseases by utilizing OMIM.

## Figures and Tables

**Table 1:** Demographics of participants with at least one sample (*n* = 199).

	TD ** (*n* = 115)	Non-TD (*n* = 27)	ASD (*n* = 57)	*p*-Value from Covariate-Only Model ^#^
Child sex: *n* (%)				0.04 *
Female	56 (48.7)	12 (44.4)	17 (29.8)	
Male	59 (51.3)	15 (55.6)	40 (70.2)	
Gestational age at delivery (weeks) Mean (SD)	38.84 (1.39)	39.29 (1.09)	39.33 (1.18)	0.06 *
Parity: *n* (%)				0.80
1 or less	53 (46.1)	12 (44.4)	22 (38.6)	
2 or more	62 (53.9)	15 (55.6)	35 (61.4)	
Child race: *n* (%)				0.50 ^†^
White	71 (61.7)	15 (55.6)	37 (64.9)	
Black/African American	0 (0.0)	2 (7.4)	5 (8.8)	
Asian	18 (15.7)	3 (11.1)	4 (7.0)	
Pacific Islander	1 (0.9)	0 (0.0)	0 (0.0)	
Multi-racial	25 (21.7)	7 (25.9)	11 (19.3)	
Child ethnicity: *n* (%)				0.93
Hispanic/Latinx	38 (33.0)	10 (37.0)	21 (36.8)	
Not Hispanic/Latinx	77 (67.0)	17 (63.0)	36 (63.2)	
Child’s year of birth: mean (SD)	2011.83 (2.20)	2012.44 (2.06)	2012.02 (1.99)	0.41
Mother’s age at time of birth: mean (SD)	34.41 (4.91)	33.44 (4.91)	34.18 (5.16)	0.66
Maternal BMI before pregnancy: mean (SD)	26.12 (5.63)	29.31 (9.71)	28.67 (7.83)	0.01 *
Maximum parental education: *n* (%)				0.69
High school or less	7 (6.1)	1 (3.7)	4 (7.1)	
Some college/2 y vocational degree	29 (25.2)	12 (44.4)	20 (35.7)	
Bachelor’s degree	43 (37.4)	7 (25.9)	20 (35.7)	
MS’s/Doctorate/Prof. degree	36 (31.3)	7 (25.9)	12 (21.4)	
Missing	0	0	1	
Homeowner status: *n* (%)				0.32
No	47 (41.2)	12 (46.2)	30 (54.5)	
Yes	67 (58.8)	14 (53.8)	25 (45.5)	
Missing	1	1	2	
Maternal vitamin use at 1 m of pregnancy: *n* (%)				0.33
No	53 (46.5)	12 (46.2)	34 (60.7)	
Yes	61 (53.5)	14 (53.8)	22 (39.3)	
Missing	1	1	1	

* The covariate has a *p*-value < 0.20 in the covariate-only models. ^†^ *p*-value is based on race being included in the model as white vs. others. ** Children were clinically classified as typically developing (TD) based on clinical assessments and scores within the normal range on standardized instruments (ADOS and MSEL). This is not considered a definitive diagnosis, especially at age 3 years, given the possibility that later they might develop conditions that present at older ages (ADHD, OCD, schizophrenia, among others). ^#^ *p*-values for test of differences across all outcomes in one covariate baseline logit model.

**Table 2:** Selected results from significant (*p* < 0.05) single-metabolite analyses of log2-transformed, centered, and scaled single-metabolite analyses using annotated metabolites identified from the negative mode in maternal plasma (38 metabolites), in discrete logistic models comparing odds of ASD or odds of Non-TD with TD used as the reference category in each case associated with a 2-fold change in the standardized abundance of a given metabolomic feature, controlling for child’s sex, race, and gestational age in weeks, and maternal pre-pregnancy BMI (*n* = 184).

Metabolite	Outcome **	OR (95% CI)	Raw *p*-Value	FDR
Norvaline	ASD	1.53 (1.07, 2.26)	0.025	0.512
Mevalonic acid	ASD	1.68 (1.09, 2.76)	0.027	0.512
Docosahexaenoic acid	Non-TD	0.28 (0.14, 0.50)	<0.001	0.003 *
Palmitic acid	Non-TD	0.38 (0.20, 0.65)	0.001	0.020 *
Palmitoleic acid	Non-TD	0.46 (0.27, 0.73)	0.002	0.023 *
Gamma-linolenic acid	Non-TD	0.46 (0.27, 0.74)	0.003	0.023 *
Myristic acid	Non-TD	0.49 (0.29, 0.78)	0.004	0.023 *
Stearic acid	Non-TD	0.42 (0.22, 0.73)	0.004	0.023 *
Heptadecanoic acid	Non-TD	0.47 (0.26,0.77)	0.005	0.023 *
Linoleic acid	Non-TD	0.48 (0.27, 0.78)	0.005	0.023 *
Gulonolactone	Non-TD	2.70 (1.18, 7.57)	0.038	0.143
Citrulline	Non-TD	0.64 (0.41, 0.97)	0.04	0.143
Hypoxanthine	Non-TD	0.64 (0.40, 0.99)	0.046	0.143
Glyceric acid	Non-TD	0.63 (0.40, 0.99)	0.046	0.143

* indicate FDR *p*-value < 0.05. ** Numbers of subjects in each group: ASD (*n* = 57), Non-TD (*n* = 27), and TD (*n* = 115).

## Data Availability

De-identified metabolomics data generated through this work are available through NDAR and CHEAR/HHEAR.

## Supplementary Materials

The following are available online at https://www.mdpi.com/article/10.3390/metabo11080545/s1, Figure S1: Volcano plot of maternal plasma full feature positive mode raw *p*-values in associations with risk of ASD (A) and Non-TD (B) compared to TD (6252 metabolites). Volcano plots with FDR-corrected *p*-values in associations with ASD (C) and Non-TD (D) are also provided, Figure S2: Volcano plot of maternal plasma full feature negative mode raw *p*-values in associations with risk of ASD (A) and Non-TD (B) compared to TD (4510 metabolites). Volcano plots with FDR-corrected *p*-values in associations with ASD (C) and Non-TD (D) are also provided, Figure S3: Volcano plot of cord plasma full feature positive mode raw *p*-values in associations with risk of ASD (A) and Non-TD (B) compared to TD (6276 metabolites). Volcano plots with FDR-corrected *p*-values in associations with ASD (C) and Non-TD (D) are also provided, Figure S4: Volcano plot of cord plasma full feature negative mode raw *p*-values in associations with risk of ASD (A) and Non-TD (B) compared to TD (4507 metabolites). Volcano plots with FDR-corrected *p*-values in associations with ASD (C) and Non-TD (D) are also provided, Figure S5: Results from principal component (PC) regression of diagnostic group on PCs identified using a PCA of full feature metabolites from the positive mode in maternal plasma, controlling for child’s sex, race, and gestational age in weeks, and maternal pre-pregnancy BMI (*n* = 184). (A) PC13 was significantly associated with ASD compared to TD (β = −4.16, *p =* 0.005). (B) Top contributing components of PC13 are shown below, Figure S6: Results from principal component (PC) regression of diagnostic group on PCs identified using a PCA of full feature metabolites from the negative mode of maternal plasma, controlling for child’s sex, race, and gestational age in weeks, and maternal pre-pregnancy BMI (*n* = 184). (A) PC1 was significantly associated with Non-TD compared to TD (β = −12.6, *p =* 0.001) and PC6 and PC12 were significantly associated with ASD compared to TD (β = 4.51, *p =* 0.020 and β = −2.89, *p =* 0.037). (B) Top contributing components of PC1 and PC6 are shown below, Figure S7: Results from principal component (PC) regression of diagnostic group on PCs identified using a PCA of full feature metabolites from the negative mode in cord plasma, controlling for child’s sex, race, and gestational age in weeks, and maternal pre-pregnancy BMI (*n* = 142). (A) PC12 was significantly associated with ASD compared to TD (β = 2.77, *p =* 0.045). (B) Top contributing components of PC12 are shown below. The dotted red line indicated the threshold 1/p where p is the number of predictors, Figure S8: Volcano plots of annotated metabolites identified from the positive mode of maternal plasma with raw *p*-values in associations with ASD (A) and Non-TD (B) compared to TD and FDR-corrected *p*-values in associations with ASD (C) and Non-TD (D) diagnoses, Figure S9: Volcano plots of annotated metabolites identified from the negative mode of maternal plasma with raw *p*-values in associations with ASD (A) and Non-TD (B) compared to TD and FDR-corrected *p*-values in associations with ASD (C) and Non-TD (D) diagnoses, Figure S10: Volcano plots of annotated metabolites identified from the positive mode of cord plasma with raw *p*-values in associations with ASD (A) and Non-TD (B) compared to TD and FDR-corrected *p*-values in associations with ASD (C) and Non-TD (D) diagnoses, Figure S11: Volcano plots of annotated metabolites identified from the negative mode of cord plasma with raw *p*-values in associations with ASD (A) and Non-TD (B) compared to TD and FDR-corrected *p*-values in associations with ASD (C) and Non-TD (D) diagnoses, Figure S12: Results from principal component (PC) regression of diagnostic group on PCs identified from a PCA of annotated metabolites from the positive mode in maternal plasma, controlling for child’s sex, race, and gestational age in weeks, and maternal pre-pregnancy BMI (*n* = 142). PC2 and PC13 were significantly associated with Non-TD compared to TD (β = 0.88, *p =* 0.017 and β = 0.55, *p =* 0.019, respectively). (A) PC8 was significantly associated with ASD compared to TD (β = 0.41, *p =* 0.052). (B) Top contributing components of PC2, PC8, and PC13 are shown below, Figure S13: Results from principal component (PC) regression of diagnostic group on PCs identified from a PCA of annotated metabolites from the negative mode of maternal plasma, controlling for child’s sex, race, and gestational age in weeks, and maternal pre-pregnancy BMI. (A) PC1 and PC8 were significantly associated with Non-TD compared to TD (β = −2.16, *p ≤* 0.001 and β = 0.60, *p =* 0.017, respectively). (B) Top contributing components of PC1 and PC8 are shown below, Figure S14: Results from principal component (PC) regression of diagnostic group on PCs from a PCA of annotated metabolites from the positive mode of cord plasma, controlling for child’s sex, race, and gestational age in weeks, and maternal pre-pregnancy BMI. (A) PC4, PC5, and PC11 were significantly associated with Non-TD compared to TD (β = −0.98, *p =* 0.010; β = 0.76, *p =* 0.046; and β = −0.61, *p =* 0.050, respectively). (B) Top contributing components of PC4 and PC5 are shown below, Figure S15: Results from principal component (PC) regression of diagnostic group on PCs from a PCA of annotated metabolites from the negative mode of cord plasma, controlling for child’s sex, race, and gestational age in weeks, and maternal pre-pregnancy BMI. (A) PC6 was significantly associated with Non-TD compared to TD (β = −1.16, *p =* 0.001). (B) Top contributing components of PC6 are shown below, Table S1: Number of metabolites identified within each matrix and mode after removing metabolites with <80% detection, Table S2: Odds Ratios (OR) and 95% Confidence Intervals (CI) from WQSRS regression of Non-TD and the full feature negative maternal plasma metabolite mixture, controlling for child’s sex, race, and gestational age in weeks, and maternal pre-pregnancy BMI (*n* = 184). 30,000 subsets with 10 metabolites per subset were used to estimate weights of each metabolite, Table S3: Selected results from significant (*p* < 0.05) single-metabolite analyses of log2-transformed, centered, and scaled single metabolite using annotated metabolites identified from the positive mode in maternal plasma (40 metabolites), in discrete logistic models comparing odds of ASD or odds of Non-TD, with TD used as the reference category in each case associated with a 2-fold change in the standardized abundance of a given metabolomic feature, controlling for child’s sex, race, and gestational age in weeks, and maternal pre-pregnancy BMI (*n* = 184), Table S4: Selected results from significant (*p* < 0.05) single-metabolite analyses of log2-transformed, centered, and scaled single metabolite using annotated metabolites identified from the positive mode in maternal plasma (40 metabolites), in discrete logistic models comparing odds of ASD or odds of Non-TD, with TD used as the reference category in each case associated with a 2-fold change in the standardized abundance of a given metabolomic feature, controlling for child’s sex, race, and gestational age in weeks, and maternal pre-pregnancy BMI (*n* = 184), Table S5: Selected results from significant (*p* < 0.05) single-metabolite analyses of log2-transformed, centered, and scaled single-metabolite analyses of annotated metabolites from the positive mode in cord plasma (37 metabolites), in discrete logistic models comparing odds of ASD or odds of Non-TD with TD used as the reference category in each case associated with a 2-fold change in the standardized abundance of a given metabolomic feature, controlling for child’s sex, race, and gestational age in weeks, and maternal pre-pregnancy BMI (*n* = 142), Table S6: Selected results from significant (*p* < 0.05) single-metabolite analyses of log2-transformed, centered, and scaled single-metabolite analyses using annotated metabolites identified from the negative mode in cord plasma (42 metabolites), in discrete logistic models comparing odds of ASD or odds of Non-TD with TD used as the reference category in each case associated with a 2-fold change in the standardized abundance of a given metabolomic feature, controlling for child’s sex, race, and gestational age in weeks, and maternal pre-pregnancy BMI (*n* = 142), Table S7: Results from rsWQS for each mode/matrix, outcome, and constraint using the annotated set of metabolites. Results are not reported for NT vs. TD for both constraints using metabolites identified in positive cord blood due to poor model fit, Table S8: Results from WQS_RS_ regression of Non-TD and the annotated negative maternal plasma metabolite mixture controlling for child’s sex, race, and gestational age in weeks, and maternal pre-pregnancy BMI (*n* = 184). 5000 subsets with 6 metabolites per subset were used to estimate weights of each metabolite. The top 10 metabolites with the highest weight contributions, representing 69.7% of the overall index, are displayed.
