# Supplementary material for: Maternal Plasma Metabolic Profile Demarcates a Role for Neuroinflammation in Non-Typical Development of Children
Source: Metabolites. 2021 Aug 18;11(8):545. doi: 10.3390/metabo11080545 (PMC8400060; doi:10.3390/metabo11080545)

Table S1: Number of metabolites identified within each matrix and mode after removing metabolites with <80% detection.

	Matrix	Mode	N samples	N metabolites
Full feature	Prenatal maternal plasma	Positive	184	6252
		Negative	184	4510
	Cord plasma	Positive	142	6276
		Negative	142	4507
Annotated features only	Prenatal maternal plasma	Positive	184	40
		Negative	184	38
	Cord plasma	Positive	142	37
		Negative	142	42

Table S2: Odds Ratios (OR) and 95% Confidence Intervals (CI) from WQS_{RS} regression of Non-TD and the full feature negative maternal plasma metabolite mixture, controlling for child's sex, race, and gestational age in weeks, and maternal pre-pregnancy BMI (N=184). Subsets (30,000) with 10 metabolites per subset were used to estimate weights of each metabolite. The top 10 metabolites with the highest weight contributions, representing 1.2% of the overall index, are displayed.

OR (95% CI)	0.25 (0.07, 0.90)
p-value	0.034*
Metabolite	Weight (%)
505.3345_207.1	0.135
352.2856_181.5	0.123
635.5243_246.4	0.122
466.9357_44.4	0.117
291.0057_55.1	0.117
319.2222_258.8	0.115
496.9262_56.5	0.115
87.474_36.8	0.113
592.9459_56.1	0.112
445.3169_177.1	0.112

* indicates *p*-value <0.05

Table S3: Results from rsWQS for each mode/matrix, outcome, and constraint, using the full feature set of metabolites.

Mode/Matrix	Outcome	Constraint	OR (95% CI)	*p*-value
Positive Plasma	ASD	Positive	1.89 (0.47, 7.51)	0.368
	ASD	Negative	2.93 (0.52, 16.41)	0.222
	Non-TD	Positive	0.09 (0.01,1.62)	0.104
	Non-TD	Negative	2.56 (0.71, 9.15)	0.151
Negative Plasma	ASD	Positive	1.60 (0.33, 7.74)	0.557
	ASD	Negative	0.91 (0.29, 2.83)	0.865
	Non-TD	Positive	0.47 (0.15, 1.45)	0.189
	Non-TD	Negative	0.25 (0.07, 0.90)	0.034*
Positive Cord Blood	ASD	Positive	1.40 (0.08, 25.27)	0.821
	ASD	Negative	1.99 (0.26, 14.96)	0.505
	Non-TD	Positive	0.96 (0.08, 11.87)	0.977
	Non-TD	Negative	1.17 (0.32, 4.33)	0.817
Negative Cord Blood	ASD	Positive	0.35 (0.05, 2.54)	0.299
	ASD	Negative	0.63 (0.12, 3.32)	0.583
	Non-TD	Positive	1.71 (0.08, 36.26)	0.732
	Non-TD	Negative	2.71 (0.22, 33.03)	0.434

* Indicates *p*-value < 0.05.

Table S4: Selected results from significant (*p*<0.05) single metabolite analyses of log2 transformed, centered, and scaled single metabolite using annotated metabolites identified from the positive mode in maternal plasma (40 metabolites), in discrete logistic models comparing odds of ASD or odds of Non-TD, with TD used as the reference category in each case associated with a 2-fold change in the standardized abundance of a given metabolomic feature, controlling for child's sex, race, and gestational age in weeks, and maternal pre-pregnancy BMI (N=184).

Metabolite	Outcome	OR (95% CI)	Raw *p*-value	False Discovery Rate
O-Phosphoethanolamine	ASD	1.67 (1.11, 2.61)	0.019	0.360
Indole	ASD	1.51 (1.05, 2.25)	0.032	0.360
Betaine	ASD	1.50 (1.05, 2.21)	0.032	0.360
Serine	ASD	1.93 (1.13, 3.93)	0.036	0.360
N-Acetylglutamic acid	Non-TD	0.56 (0.36, 0.86)	0.009	0.210
Citrulline	Non-TD	0.51 (0.29, 0.83)	0.010	0.210
Acetylcarnitine	Non-TD	0.55 (0.31, 0.91)	0.027	0.341
Cystathionine	Non-TD	0.61 (0.38, 0.96)	0.034	0.341

Table S5: Selected results from significant (p<0.05) single metabolite analyses of log2 transformed, centered, and scaled single metabolite analyses of annotated metabolites from the positive mode in cord plasma (37 metabolites), in discrete logistic models comparing odds of ASD or odds of Non-TD with TD used as the reference category in each case associated with a 2-fold change in the standardized abundance of a given metabolomic feature, controlling for child's sex, race, and gestational age in weeks, and maternal pre-pregnancy BMI (N=142).

Metabolite	Outcome	OR (95% CI)	Raw *p*-value	False Discovery Rate
12-Hydroxydodecanoic acid	ASD	0.60 (0.38, 0.89)	0.016	0.567
Indoleacetic acid	ASD	1.55 (1.06, 2.38)	0.031	0.567
N-Acetyl-Leucine	Non-TD	2.30 (1.23, 4.63)	0.013	0.379
2′,4′-Dihydroxyacetophenone	Non-TD	0.58 (0.35, 0.92)	0.020	0.379
Glutamic acid	Non-TD	0.48 (0.22, 0.96)	0.046	0.571

Table S6: Selected results from significant (p<0.05) single metabolite analyses of log2 transformed, centered, and scaled single metabolite analyses using annotated metabolites identified from the negative mode in cord plasma (42 metabolites), in discrete logistic models comparing odds of ASD or odds of Non-TD with TD used as the reference category in each case associated with a 2-fold change in the standardized abundance of a given metabolomic feature, controlling for child's sex, race, and gestational age in weeks, and maternal pre-pregnancy BMI (N=142).

Metabolite	Outcome	OR (95% CI)	Raw *p*-value	False Discovery Rate
Pyrrolidonecarboxylic acid	Non-TD	0.38 (0.15, 0.82)	0.024	0.662

Table S7: Results from rsWQS for each mode/matrix, outcome, and constraint using the annotated set of metabolites. Results are not reported for NT vs TD for both constraints using metabolites identified in positive cord blood due to poor model fit.

Mode/Matrix	Outcome	Constraint	OR (95% CI)	*p*-value
Positive Plasma	ASD	Positive	0.95 (0.61, 1.47)	0.807
	ASD	Negative	1.29 (0.81, 2.05)	0.283
	Non-TD	Positive	1.02 (0.52, 2.01)	0.944
	Non-TD	Negative	0.86 (0.42, 1.75)	0.679
Negative Plasma	ASD	Positive	1.14 (0.69, 1.88)	0.621
	ASD	Negative	0.91 (0.60, 1.39)	0.668
	Non-TD	Positive	1.03 (0.55, 1.94)	0.916
	Non-TD	Negative	0.56 (0.30, 1.04)	0.065
Positive Cord Blood	ASD	Positive	1.23 (0.67, 2.25)	0.513
	ASD	Negative	1.38 (0.74, 2.57)	0.318
	Non-TD	Positive	--	--
	Non-TD	Negative	--	--
Negative Cord Blood	ASD	Positive	0.91 (0.45, 1.84)	0.783
	ASD	Negative	1.46 (0.76, 2.79)	0.256
	Non-TD	Positive	1.36 (0.56, 3.27)	0.496
	Non-TD	Negative	0.73 (0.30, 1.79)	0.491

Table S8: Results from WQS_{RS} regression of Non-TD and the annotated negative maternal plasma metabolite mixture controlling for child's sex, ethnicity, and gestational age in weeks, and maternal pre-pregnancy BMI (n=184). 5,000 subsets with 6 metabolites per subset were used to estimate weights of each metabolite. The top 10 metabolites with the highest weight contributions, representing 69.7% of the overall index, are displayed.

OR (95% CI)	0.56 (0.30, 1.04)
p-value	0.065
Metabolite	Weight %
Galacturonic acid	9.82
Docosahexaenoic acid	8.52
Acetylglycine	8.41
Gamma-Linolenic acid	7.61
Ribose	7.25
Citrulline	5.93
Palmitic acid	5.74
Inosine-5′-diphosphate	5.67
Stearic acid	5.15
Hypoxanthine	5.63

Figures

Figure S1: Volcano plot of maternal plasma full feature positive mode raw *p*-values in associations with risk of ASD (A) and Non-TD (B) compared to TD (6252 metabolites). Volcano plots with FDR-corrected *p*-values in associations with ASD (C) and Non-TD (D) are also provided. The x-axis shows beta estimates (i.e. change in log odds associated with a 2-fold change in the standardized abundance of a given metabolomic feature) controlling for child's sex, ethnicity, and gestational age in weeks, and maternal pre-pregnancy BMI, while the y-axis shows -log10 FDR corrected *p*-values (N=184).

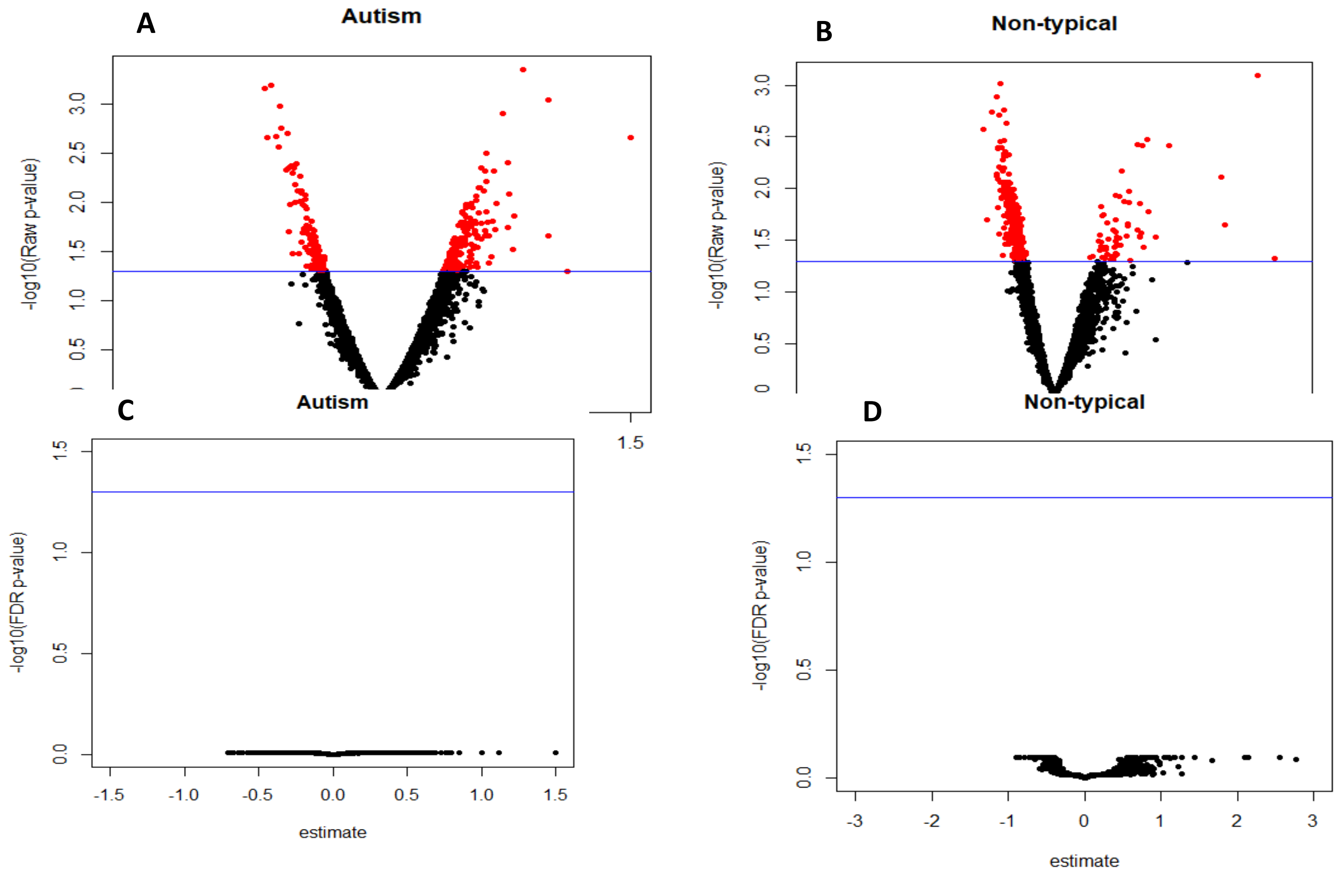

Figure S2: Volcano plot of maternal plasma full feature negative mode raw *p*-values in associations with risk of ASD (A) and Non-TD (B) compared to TD (4510 metabolites). Volcano plots with FDR-corrected *p*-values in associations with ASD (C) and Non-TD (D) are also provided. The x-axis shows beta estimates (i.e. change in log odds associated with a 2-fold change in the standardized abundance of a given metabolomic feature) controlling for child's sex, ethnicity, and gestational age in weeks, and maternal pre-pregnancy BMI, while the y-axis shows -log10 FDR corrected *p*-values (N=184).

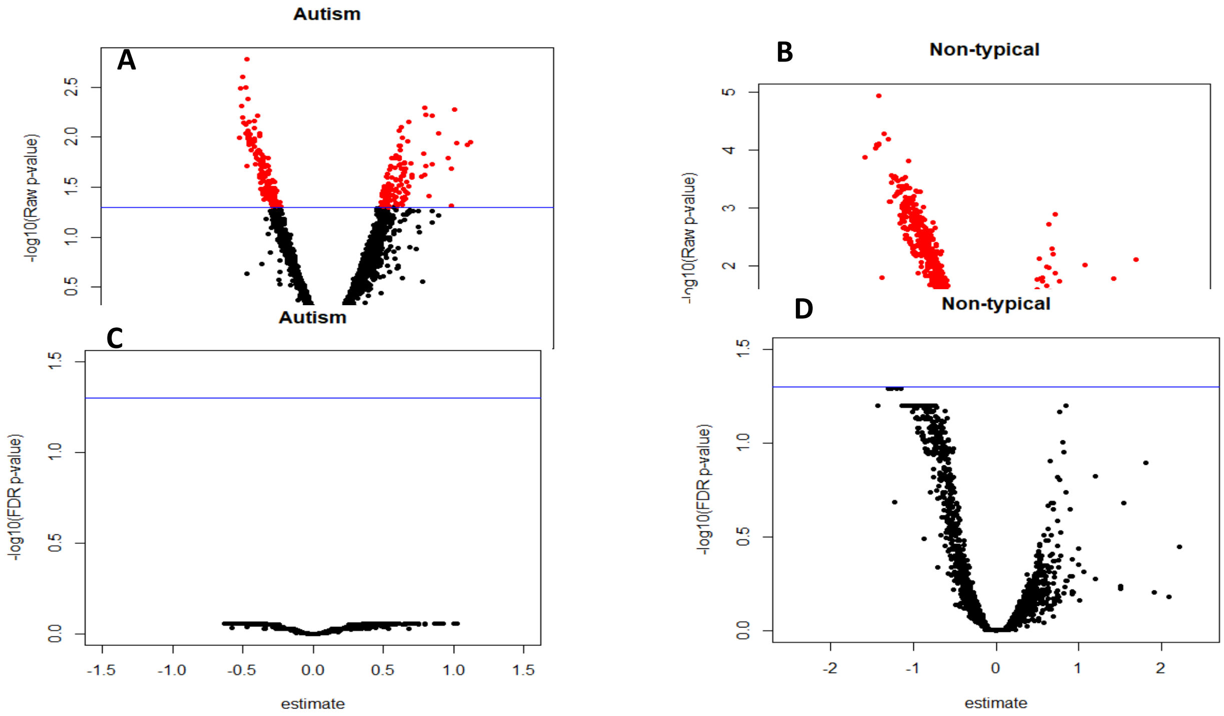

Figure S3: Volcano plot of cord plasma full feature positive mode raw *p*-values in associations with risk of ASD (A) and Non-TD (B) compared to TD (6276 metabolites). Volcano plots with FDR-corrected *p*-values in associations with ASD (C) and Non-TD (D) are also provided. The x-axis shows beta estimates (i.e. change in log odds associated with a 2-fold change in a given metabolomic feature) controlling for child's sex, race, and gestational age in weeks, and maternal pre-pregnancy BMI, while the y-axis shows -log10 FDR corrected *p*-values (N=142).

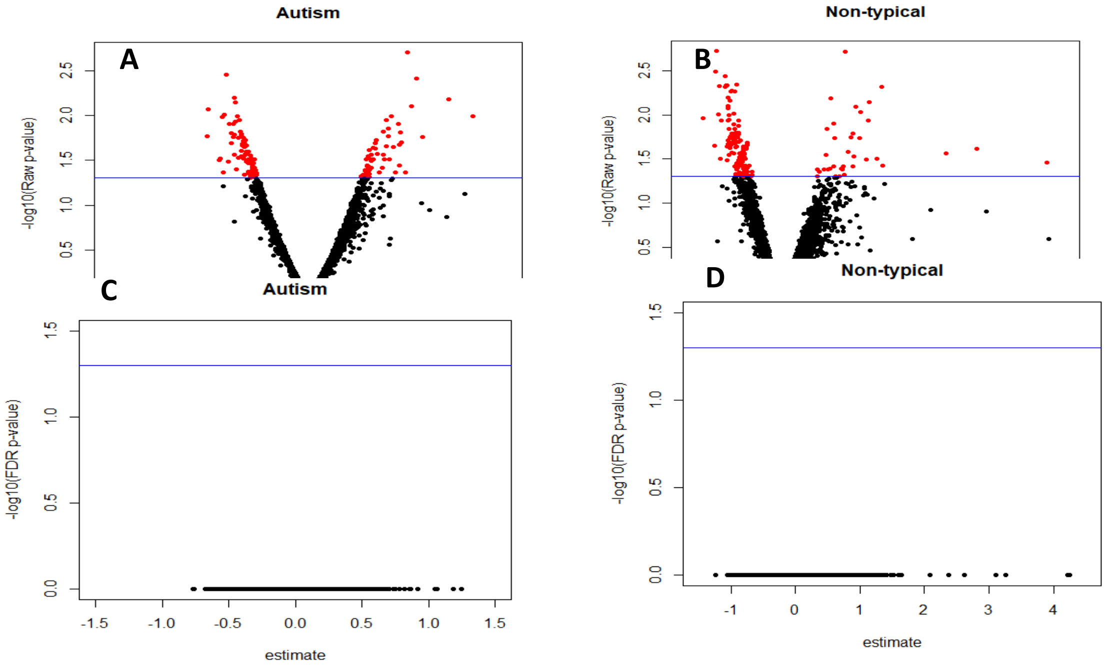

Figure S4: Volcano plot of cord plasma full feature negative mode raw *p*-values in associations with risk of ASD (A) and Non-TD (B) compared to TD (4507 metabolites). Volcano plots with FDR-corrected *p*-values in associations with ASD (C) and Non-TD (D) are also provided. The x-axis shows beta estimates (i.e. change in log odds associated with a 2-fold change in a given metabolomic feature) controlling for child's sex, race, and gestational age in weeks, and maternal pre-pregnancy BMI, while the y-axis shows -log10 FDR corrected *p*-values (N=142).

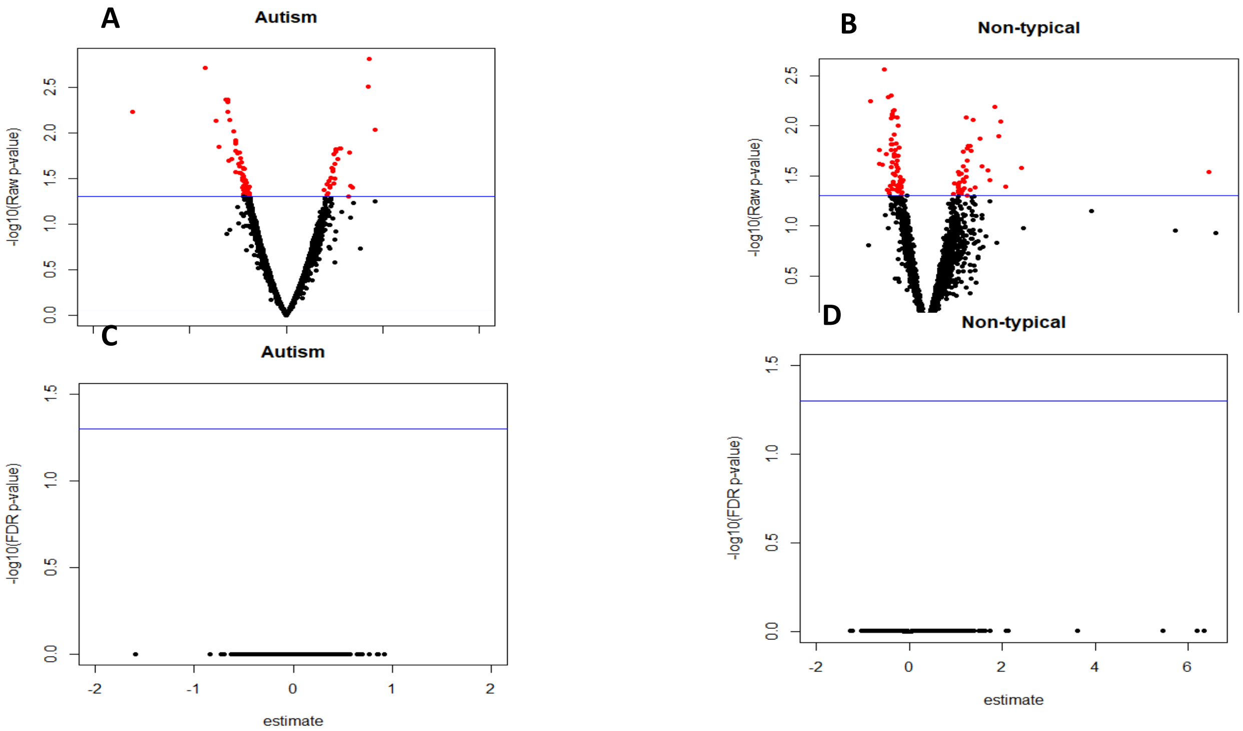

Figure S5: Results from principal component (PC) regression of diagnostic group on PCs identified using a PCA of full feature metabolites from the positive mode in maternal plasma, controlling for child's sex, race, and gestational age in weeks, and maternal pre-pregnancy BMI (N=184). (A) PC13 was significantly associated with ASD compared to TD (β=-4.16, *p*=0.005). (B) Top contributing components of PC13 are shown below. The dotted red line indicated the threshold 1/p where p is the number of predictors.

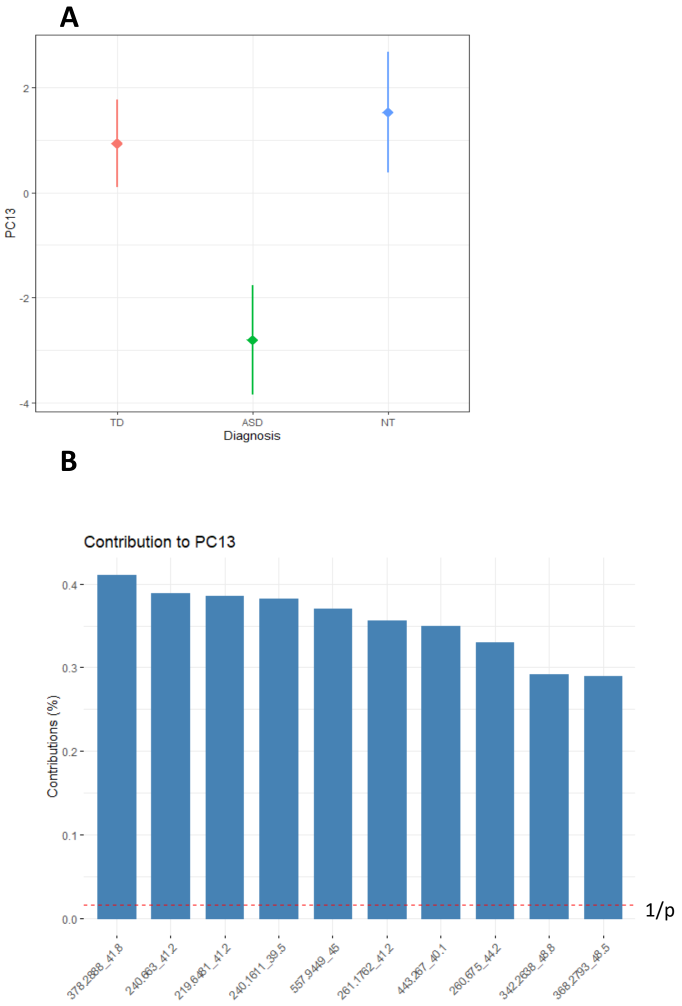

Figure S6: Results from principal component (PC) regression of diagnostic group on PCs identified using a PCA of full feature metabolites from the negative mode of maternal plasma, controlling for child's sex, race, and gestational age in weeks, and maternal pre-pregnancy BMI (N=184). (A) PC1 was significantly associated with Non-TD (NT) compared to TD (β=-12.6, *p*=0.001) and PC6 and PC12 were significantly associated with ASD compared to TD (β=4.51, *p*=0.020 and β=-2.89, *p*=0.037). (B) Top contributing components of PC1 and PC6 are shown below. The dotted red line indicated the threshold 1/p where p is the number of predictors.

A

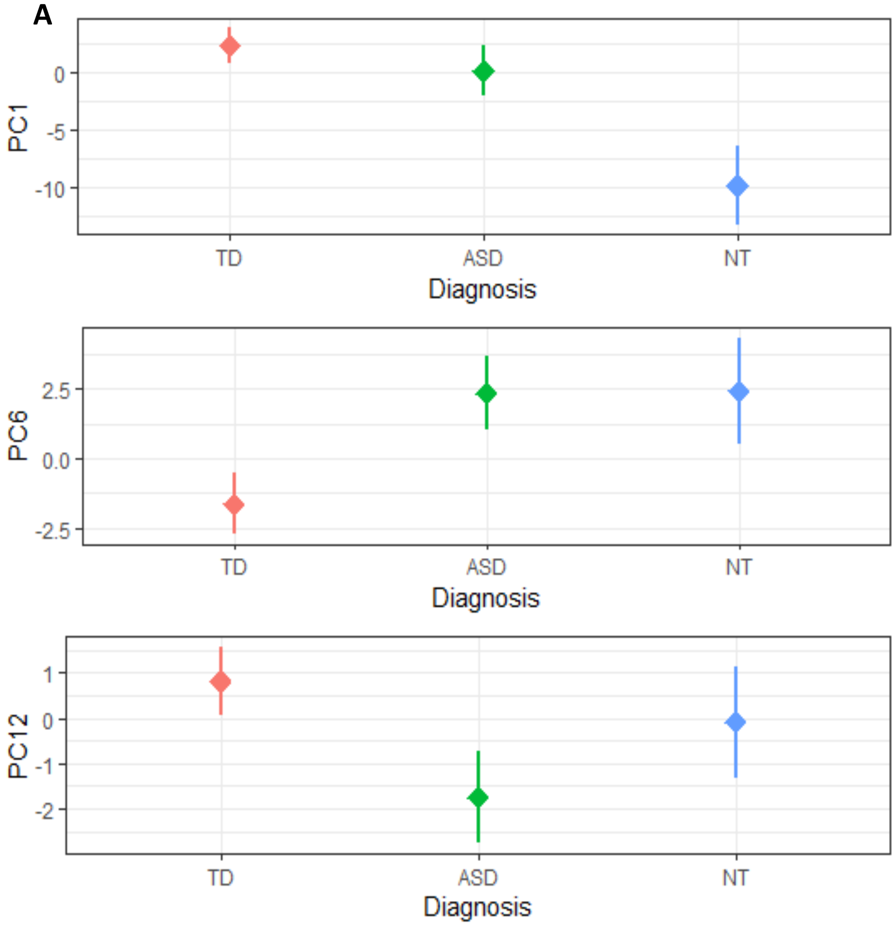

B

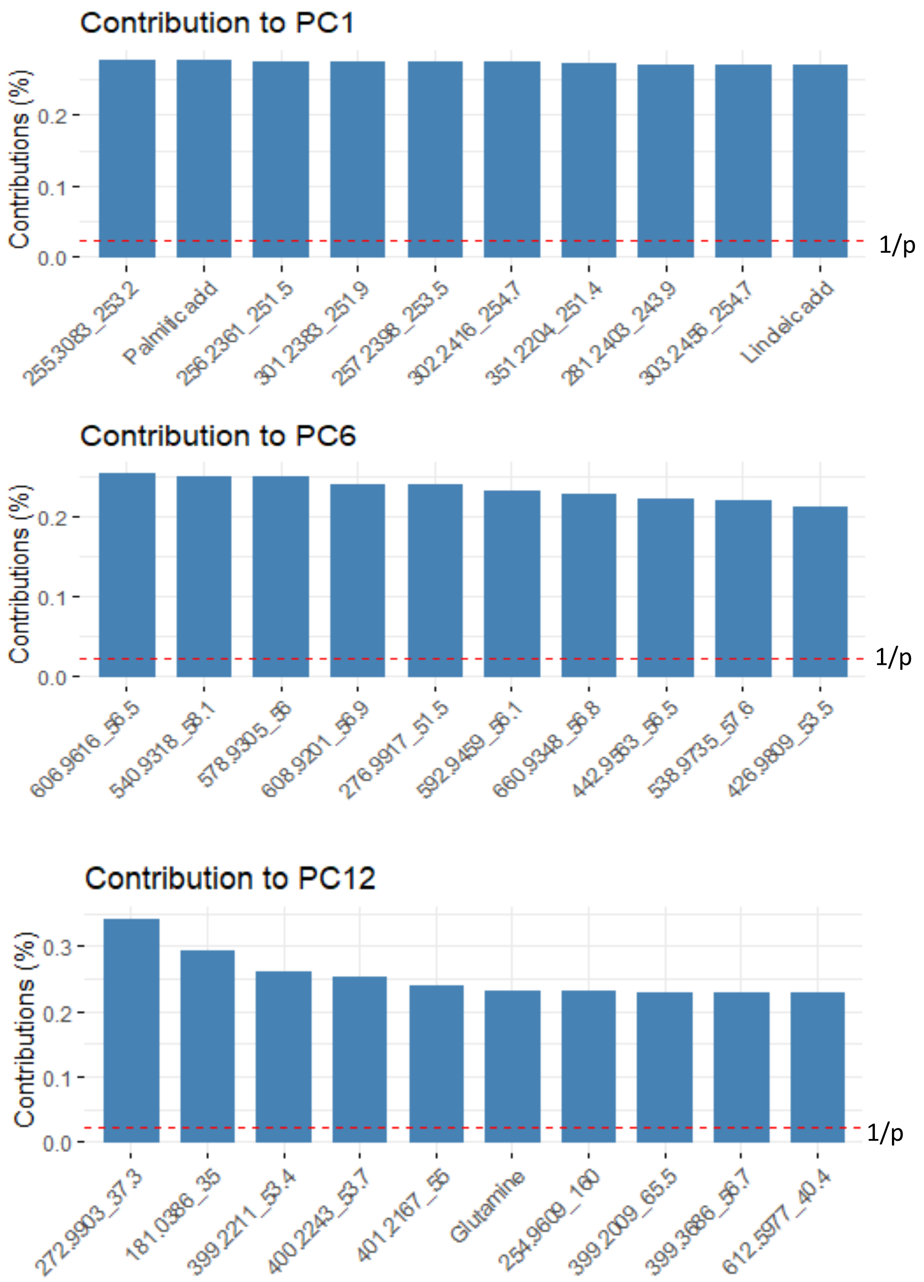

Contribution to PC1
Contributions (%)
0.0
0.1
0.2
1/p
255.3083_253.2
Palmitic acid
256.2361_251.5
301.2383_251.9
257.2398_253.5
302.2416_254.7
351.2204_251.4
281.2403_243.9
303.2456_254.7
Linoleic acid
Contribution to PC6
Contributions (%)
0.0
0.1
0.2
1/p
606.9616_56.5
540.9318_58.1
578.9305_56
608.9201_56.9
276.9917_51.5
592.9459_56.1
660.9348_56.8
442.9563_56.5
538.9735_57.6
426.9809_53.5
Contribution to PC12
Contributions (%)
0.0
0.1
0.2
0.3
1/p
272.9903_37.3
181.0386_35
399.2211_53.4
400.2243_53.7
401.2167_55
Glutamine
254.9609_160
399.2009_65.5
399.3686_56.7
612.5977_40.4

Figure S7: Results from principal component (PC) regression of diagnostic group on PCs identified using a PCA of full feature metabolites from the negative mode in cord plasma, controlling for child's sex, race, and gestational age in weeks, and maternal pre-pregnancy BMI (N=142). (A) PC12 was significantly associated with ASD compared to TD (β=2.77, p=0.045). (B) Top contributing components of PC12 are shown below. The dotted red line indicated the threshold 1/p where p is the number of predictors.

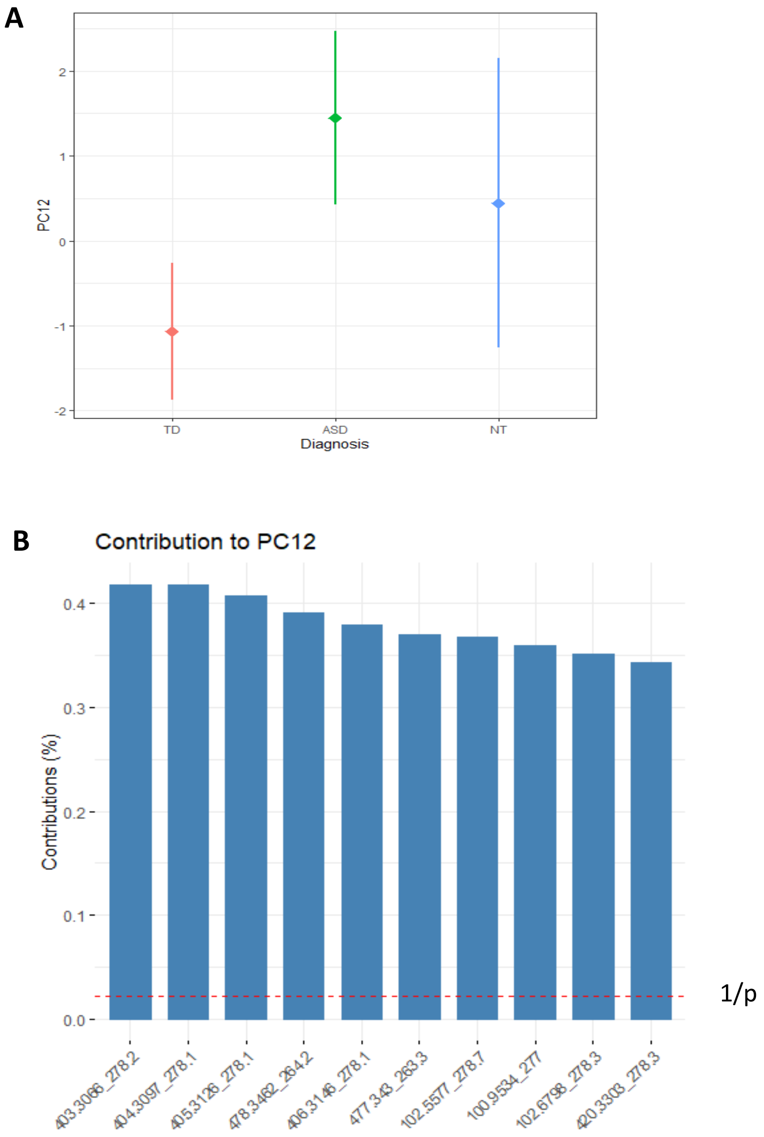

Figure S8: Volcano plots of annotated metabolites identified from the positive mode of maternal plasma with raw *p*-values in associations with ASD (A) and Non-TD (B) compared to TD and FDR-corrected *p*-values in associations with ASD (C) and Non-TD (D) diagnoses. Metabolites above the blue line indicate statistical significance. The x-axis shows beta estimates (i.e. change in log odds associated with a 2-fold change in the standardized abundance of a given metabolomic feature), while the y-axis shows either the -log10 raw *p*-values or -log10 FDR corrected *p*-values.

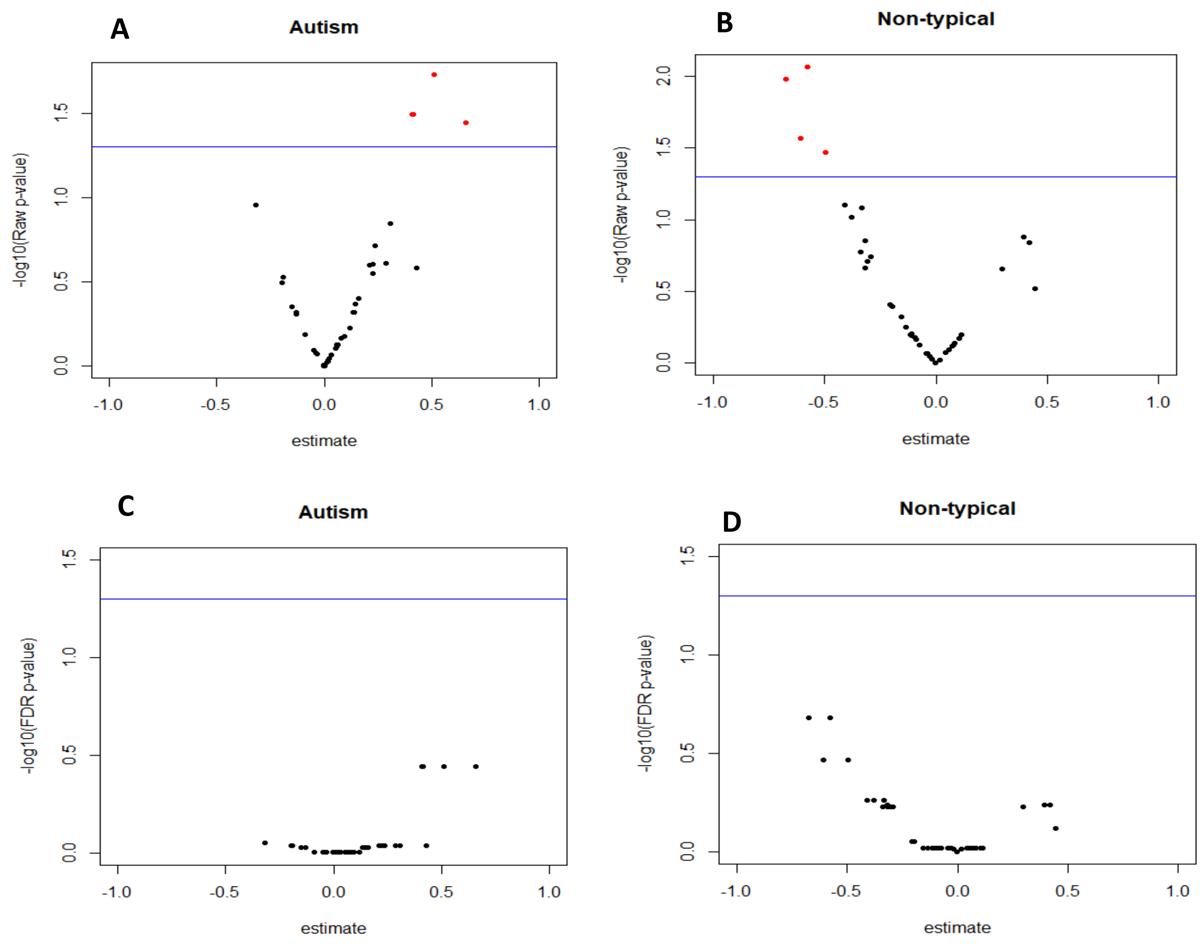

Figure S9: Volcano plots of annotated metabolites identified from the negative mode of maternal plasma with raw *p*-values in associations with ASD (A) and Non-TD (B) compared to TD and FDR-corrected *p*-values in associations with ASD (C) and Non-TD (D) diagnoses. Metabolites above the blue line indicate statistical significance. The x-axis shows beta estimates (i.e. change in log odds associated with a 2-fold change in the standardized abundance of a given metabolomic feature), while the y-axis shows either the -log10 raw *p*-values or -log10 FDR corrected *p*-values.

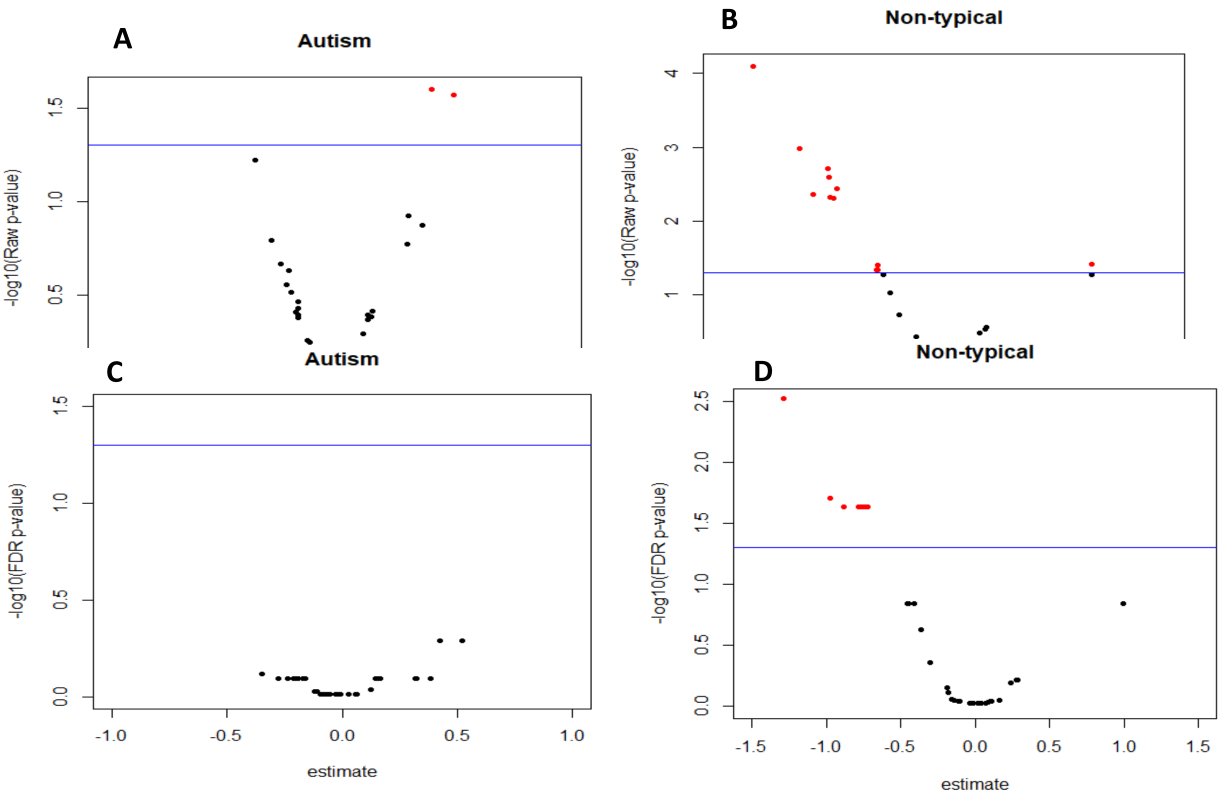

Figure S10: Volcano plots of annotated metabolites identified from the positive mode of cord plasma with raw *p*-values in associations with ASD (A) and Non-TD (B) compared to TD and FDR-corrected *p*-values in associations with ASD (C) and Non-TD (D) diagnoses. Metabolites above the blue line indicate statistical significance. The x-axis shows beta estimates (i.e. change in log odds associated with a 2-fold change in the standardized abundance of a given metabolomic feature), while the y-axis shows either the -log10 raw *p*-values or -log10 FDR corrected *p*-values.

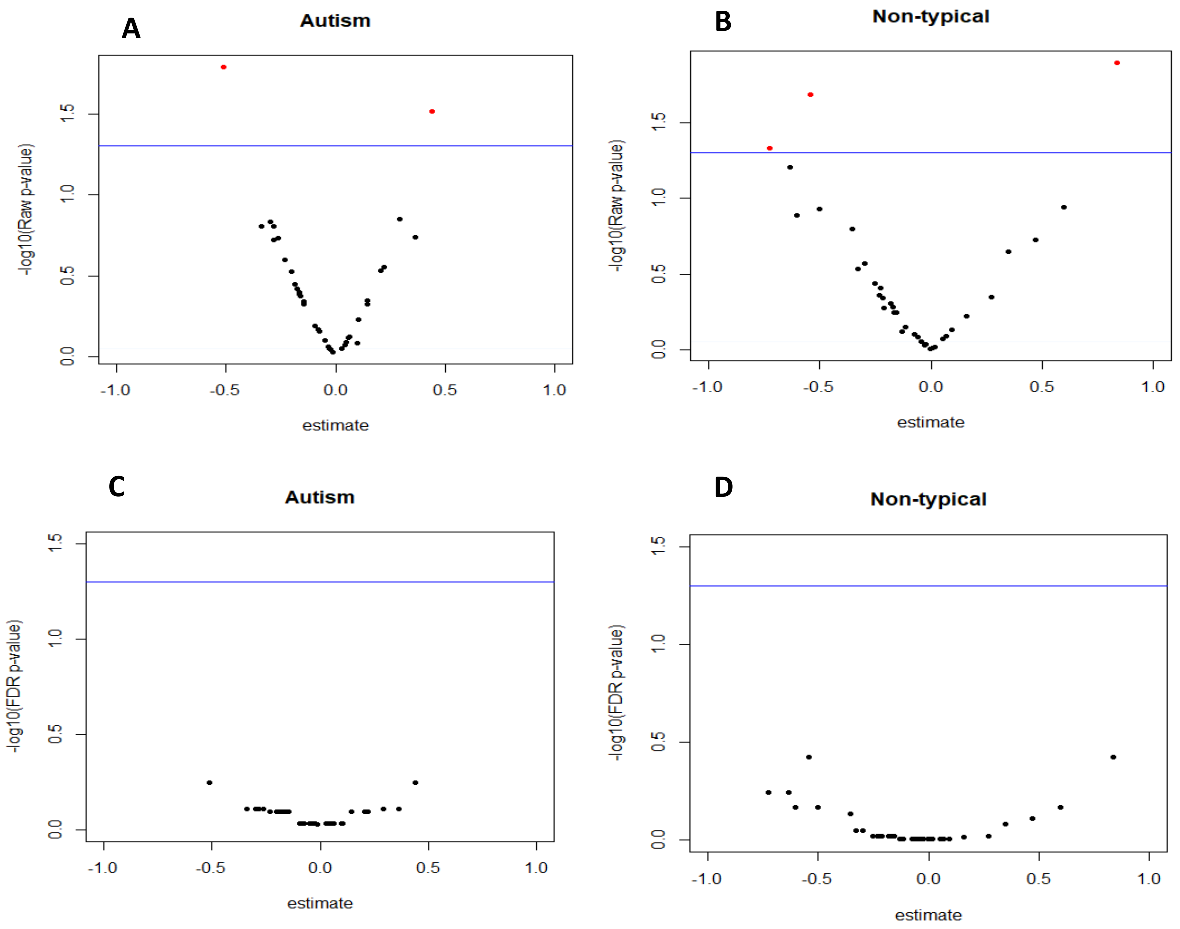

Figure S11: Volcano plots of annotated metabolites identified from the negative mode of cord plasma with raw *p*-values in associations with ASD (A) and Non-TD (B) compared to TD and FDR-corrected *p*-values in associations with ASD (C) and Non-TD (D) diagnoses. Metabolites above the blue line indicate statistical significance. The x-axis shows beta estimates (i.e. change in log odds associated with a 2-fold change in the standardized abundance of a given metabolomic feature), while the y-axis shows either the -log10 raw *p*-values or -log10 FDR corrected *p*-values.

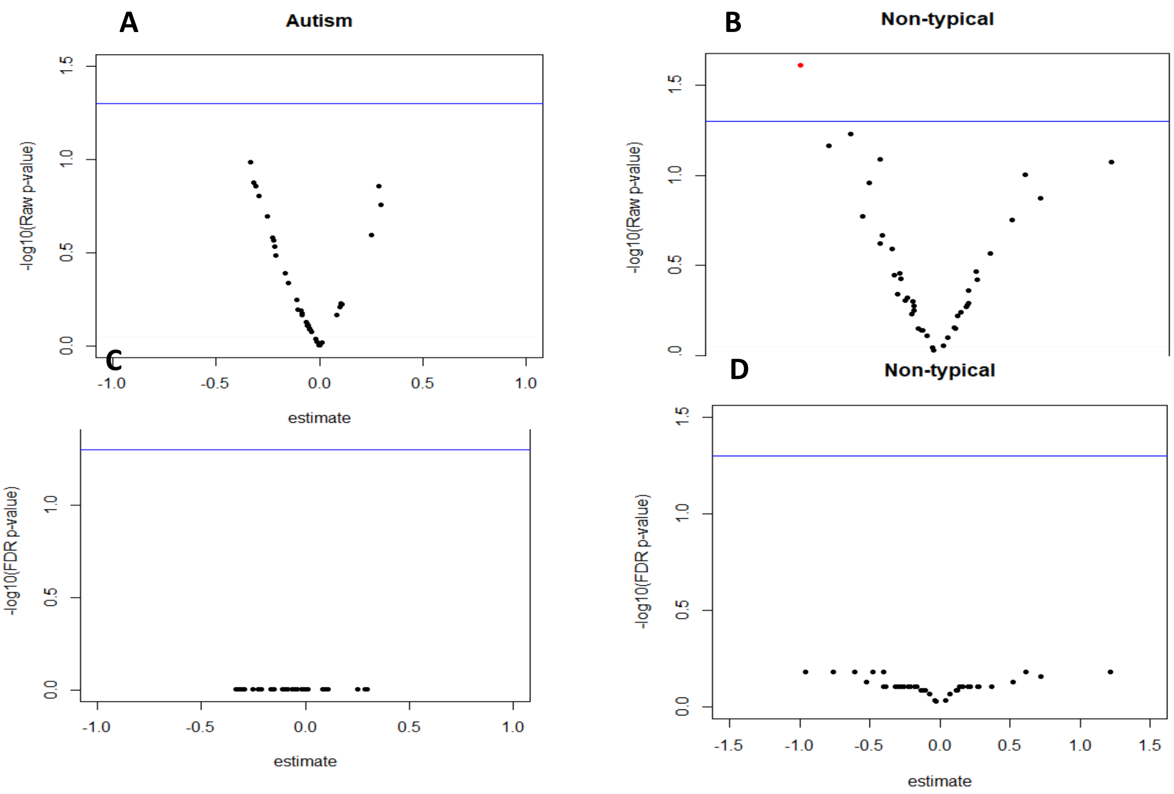

Figure S12: Results from principal component (PC) regression of diagnostic group on PCs identified from a PCA of annotated metabolites from the positive mode in maternal plasma, controlling for child's sex, race, and gestational age in weeks, and maternal pre-pregnancy BMI (N=142). PC2 and PC13 were significantly associated with Non-TD (NT) compared to TD (β=0.88, *p*=0.017 and β=0.55, *p*=0.019 respectively). (A) PC8 was significantly associated with ASD compared to TD (β=0.41, *p*=0.052). (B) Top contributing components of PC2, PC8, and PC13 are shown below. The dotted red line indicated the threshold 1/p where p is the number of predictors.

A

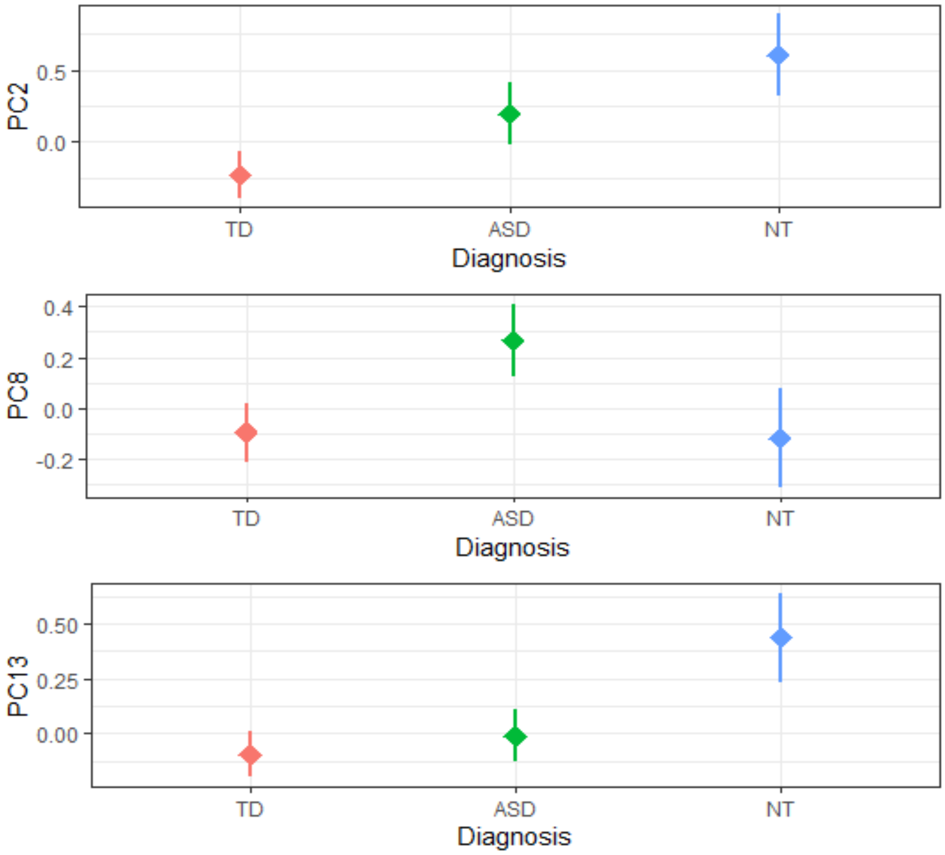

B

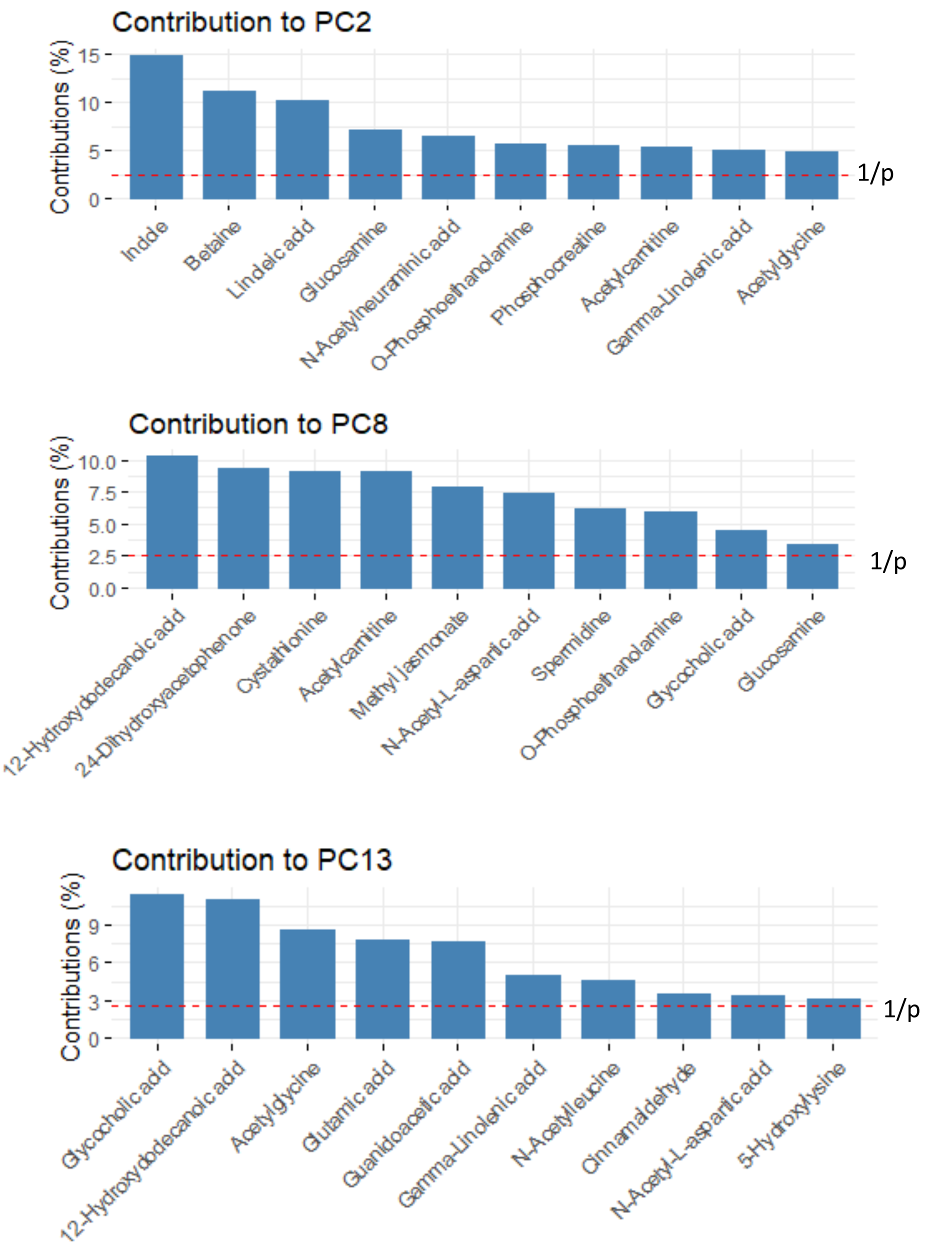
Contribution to PC2
Contributions (%)
15
10
5
0
1/p
Indole
Betaine
Linoleic acid
Glucosamine
N-Acetylneuraminic acid
O-Phosphoethanolamine
Phosphocreatine
Acetylcarnitine
Gamma-Linolenic acid
Acetylglycine
Contribution to PC8
Contributions (%)
10.0
7.5
5.0
2.5
0.0
1/p
12-Hydroxydodecanoic acid
2,4-Dihydroxyacetophenone
Cystathionine
Acetylcarnitine
Methyl jasmonate
N-Acetyl-L-aspartic acid
Spermidine
O-Phosphoethanolamine
Glycocholic acid
Glucosamine
Contribution to PC13
Contributions (%)
9
6
3
0
1/p
Glycocholic acid
12-Hydroxydodecanoic acid
Acetylglycine
Glutamic acid
Guanidoacetic acid
Gamma-Linolenic acid
N-Acetylleucine
Cinnamaldehyde
N-Acetyl-L-aspartic acid
5-Hydroxylysine

Figure S13: Results from principal component (PC) regression of diagnostic group on PCs identified from a PCA of annotated metabolites from the negative mode of maternal plasma, controlling for child's sex, race, and gestational age in weeks, and maternal pre-pregnancy BMI. (A) PC1 and PC8 were significantly associated with Non-TD (NT) compared to TD (β=-2.16, *p*=<0.001 and β=0.60, *p*=0.017 respectively). (B) Top contributing components of PC1 and PC8 are shown below. The dotted red line indicated the threshold 1/p where p is the number of predictors.

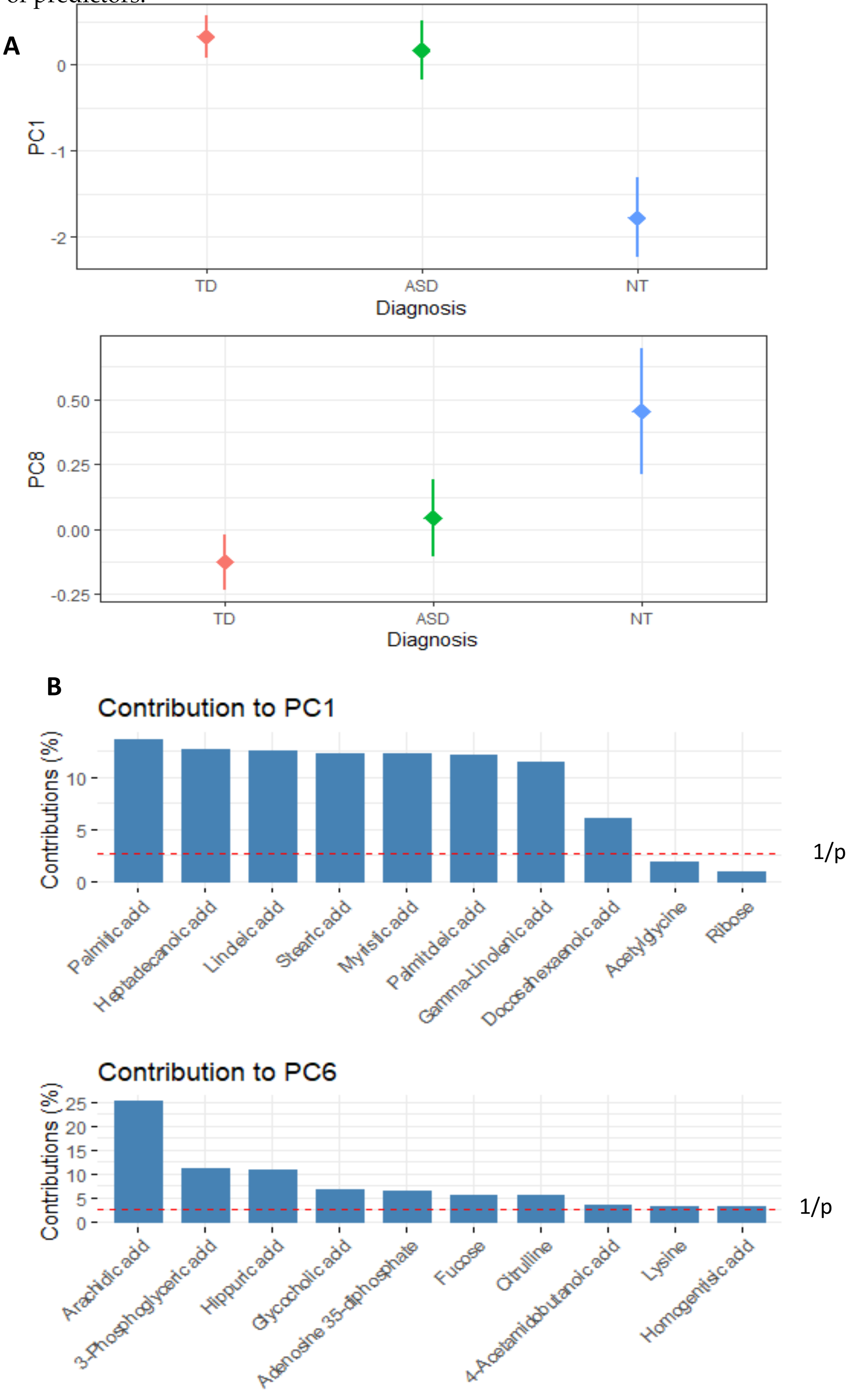

Figure S14: Results from principal component (PC) regression of diagnostic group on PCs from a PCA of annotated metabolites from the positive mode of cord plasma, controlling for child's sex, race, and gestational age in weeks, and maternal pre-pregnancy BMI. (A) PC4, PC5, and PC11 were significantly associated with Non-TD (NT) compared to TD (β=-0.98, *p*=0.010; β=0.76, *p*=0.046; and β=-0.61, *p*=0.050 respectively). (B) Top contributing components of PC4 and PC5 are shown below. The dotted red line indicated the threshold 1/p where p is the number of predictors.

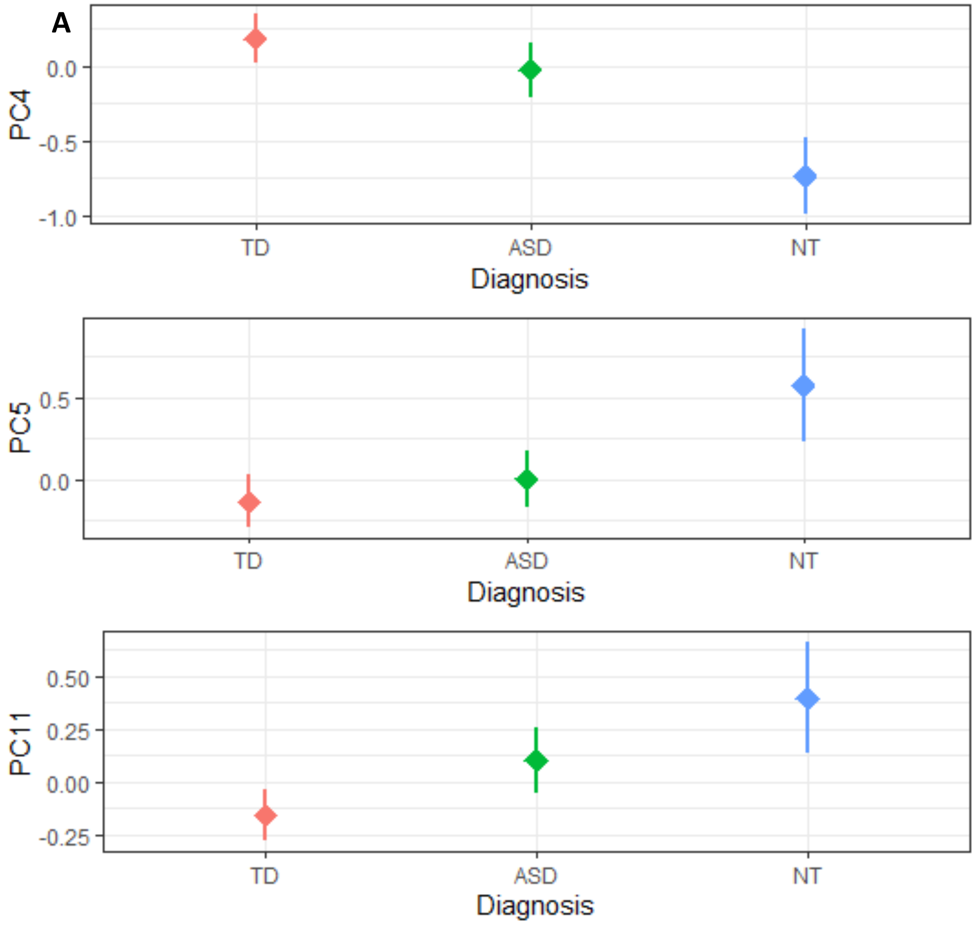

B

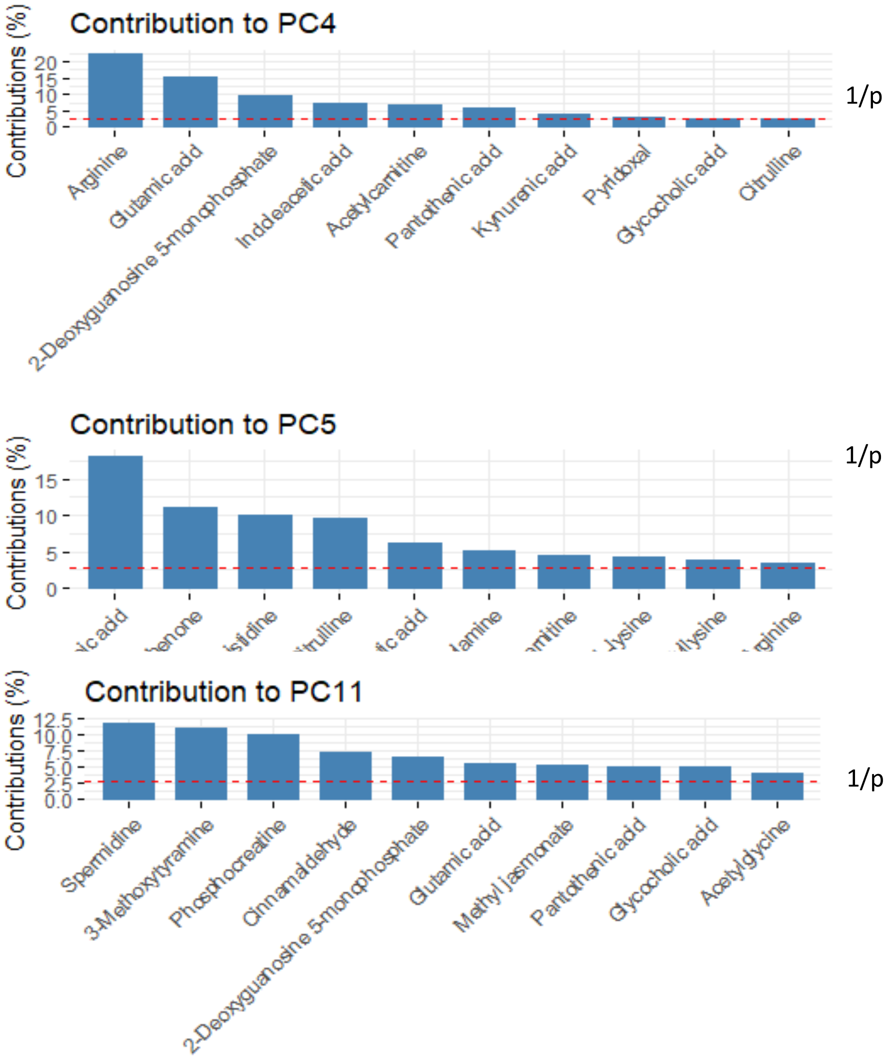
Contribution to PC4
Contributions (%)
20
15
10
5
0
Arginine
Glutamic acid
2-Deoxyguanosine 5-monophosphate
Indoleacetic acid
Acetylcarnitine
Pantothenic acid
Kynurenic acid
Pyridoxal
Glycocholic acid
Citrulline
1/p
Contribution to PC5
Contributions (%)
15
10
5
0
1/p
Contribution to PC11
Contributions (%)
12.5
10.0
7.5
5.0
2.5
0.0
Spermidine
3-Methoxytyramine
Phosphocreatine
Cinnamaldehyde
2-Deoxyguanosine 5-monophosphate
Glutamic acid
Methyl jasmonate
Pantothenic acid
Glycocholic acid
Acetylglycine
1/p

Figure S15: Results from principal component (PC) regression of diagnostic group on PCs from a PCA of annotated metabolites from the negative mode of cord plasma, controlling for child's sex, race, and gestational age in weeks, and maternal pre-pregnancy BMI. (A) PC6 was significantly associated with Non-TD (NT) compared to TD (β=-1.16, *p*=0.001). (B) Top contributing components of PC6 are shown below. The dotted red line indicated the threshold 1/p where p is the number of predictors

A

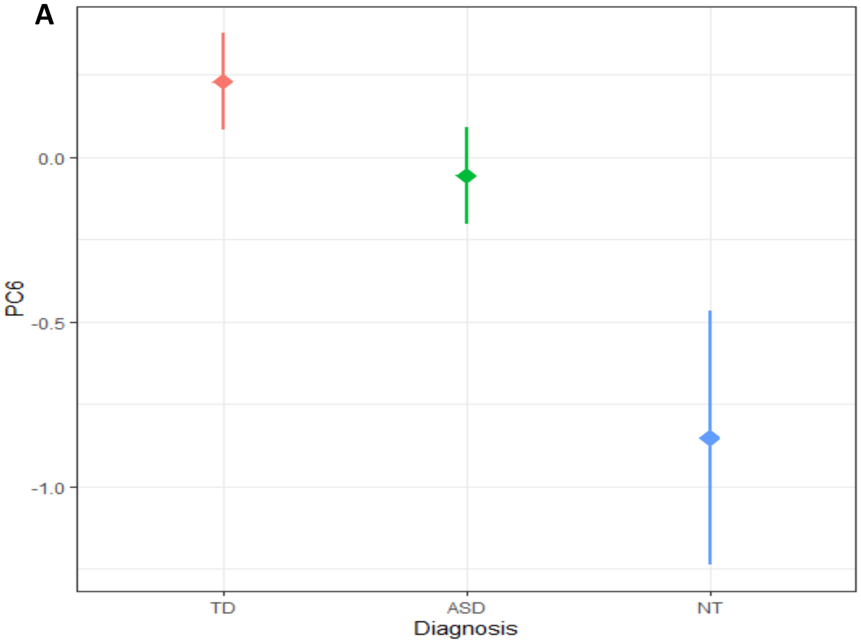

B

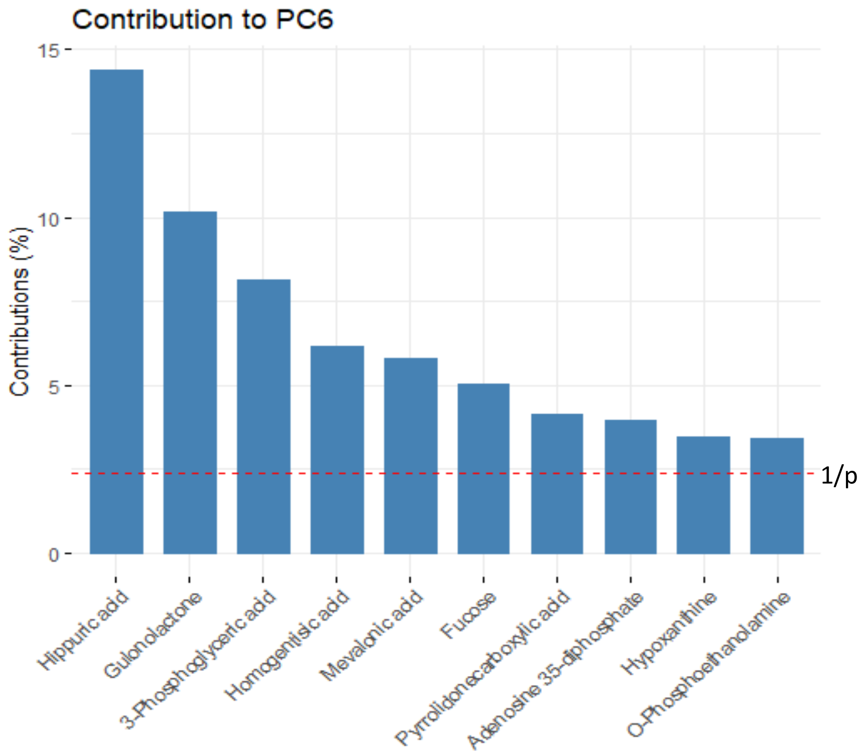

Supplement: Supplementary file 1 [file metabolites-11-00545-s001.zip › metabolites-1305661-supplementary.pdf]
